# Multilimbed membrane guanylate cyclase signaling system, evolutionary ladder

**DOI:** 10.3389/fnmol.2022.1022771

**Published:** 2023-01-05

**Authors:** Teresa Duda, Rameshwar K. Sharma

**Affiliations:** The Unit of Regulatory and Molecular Biology, Research Divisions of Biochemistry and Molecular Biology, Salus University, Elkins Park, PA, United States

**Keywords:** membrane guanylate cyclase, cyclic GMP signaling pathways, surface receptors, calcium, sensory neurons, cardiovascular, carbon dioxide, transduction modes

## Abstract

One monumental discovery in the field of cell biology is the establishment of the membrane guanylate cyclase signal transduction system. Decoding its fundamental, molecular, biochemical, and genetic features revolutionized the processes of developing therapies for diseases of endocrinology, cardio-vasculature, and sensory neurons; lastly, it has started to leave its imprints with the atmospheric carbon dioxide. The membrane guanylate cyclase does so *via* its multi-limbed structure. The inter-netted limbs throughout the central, sympathetic, and parasympathetic systems perform these functions. They generate their common second messenger, cyclic GMP to affect the physiology. This review describes an historical account of their sequential evolutionary development, their structural components and their mechanisms of interaction. The foundational principles were laid down by the discovery of its first limb, the ACTH modulated signaling pathway (the companion monograph). It challenged two general existing dogmas at the time. First, there was the question of the existence of a membrane guanylate cyclase independent from a soluble form that was heme-regulated. Second, the sole known cyclic AMP three-component-transduction system was modulated by GTP-binding proteins, so there was the question of whether a one-component transduction system could exclusively modulate cyclic GMP in response to the polypeptide hormone, ACTH. The present review moves past the first question and narrates the evolution and complexity of the cyclic GMP signaling pathway. Besides ACTH, there are at least five additional limbs. Each embodies a unique modular design to perform a specific physiological function; exemplified by ATP binding and phosphorylation, Ca^2+^-sensor proteins that either increase or decrease cyclic GMP synthesis, co-expression of antithetical Ca^2+^ sensors, GCAP1 and S100B, and modulation by atmospheric carbon dioxide and temperature. The complexity provided by these various manners of operation enables membrane guanylate cyclase to conduct diverse functions, exemplified by the control over cardiovasculature, sensory neurons and, endocrine systems.

## Introduction

Existing initially in the shadows of cyclic AMP signaling, cyclic GMP has slowly rose to prominence as a critical signaling molecule controlled by the activities of soluble and membrane guanylate cyclases (MGCs) and phosphodiesterases. After a brief outline of the chronological development of the MGC field, we focus on the uniqueness of the modular design which adds layers of complexity and enables the enzyme to perform various physiological functions.

Some of the advancements made by the authors and their collaborators are featured. The narration is partially borrowed, with appropriate citations, from the authors’ earlier reviews: (Sharma, 1978, 1985, 2002, 2010; Pugh et al., 1997; Sharma et al., 2004, 2014; Duda et al., 2005b, 2014; Sharma and Duda, 2012, 2014a,b).

## Background

Beginning with the discovery of cyclic AMP in 1958, understanding of the molecular principles of hormonal signaling began to undergo a radical change. The new paradigm was that the hormone did not directly target its signaling site. Instead it did so through its second messenger, cyclic AMP (Sutherland and Rall, 1958; Sutherland and Rall, 1960; Ariëns and Simonis, 1966; Ariëns, 1967; Robison et al., 1967).

For constructing the building blocks of this “cyclic AMP second messenger” concept Sutherland in 1971 and Rodbell and Gilamn in 1994 were awarded Nobel prizes in physiology and medicine.

In Sutherland’s original “second messenger” concept, hormone was the first messenger and its interacting cell surface receptor product, cyclic AMP, the second. Glycogen, catecholamines, and other polypeptide hormones manifested their biological activities within the cell in this manner. The principal features of this concept were envisioned to be applicable to all hormonal systems (Robison et al., 1967). The receptor was conceptualized as the critical component of a cell; there, the hormone interacted to produce a “stimulus.” The term ‘stimulus’ was equivalent to the present term ‘signal’, which according to the “second messenger” concept, meant that adenylate cyclase was the means to convert the extracellular ligand-binding signal into the production of the intracellular cyclic AMP (Ariëns and Simonis, 1966; Ariëns, 1967). Implicit in this concept was the understanding that the events leading to the transformation of the binding signal to the production of the second messenger occurred in the cell’s plasma membrane.

In the 1970’s the term “transduction” was introduced by the Rodbell’s group (Birnbaumer et al., 1970). It meant transformation of the hormonal signal into the generation of cyclic AMP. The critical molecule involved in transduction was GTP. It bound to its signaling component, termed G-protein that stimulated the next signaling component, the enzyme adenylate cyclase that catalyzed the production of cyclic AMP. The details of the last step were decoded by the groups of Gilman and Birnbaumer (reviewed in: Feder et al., 1986; Negishi et al., 1988; Hepler and Gilman, 1992; Birnbaumer and Birnbaumer, 1995).

**Figure 1:**
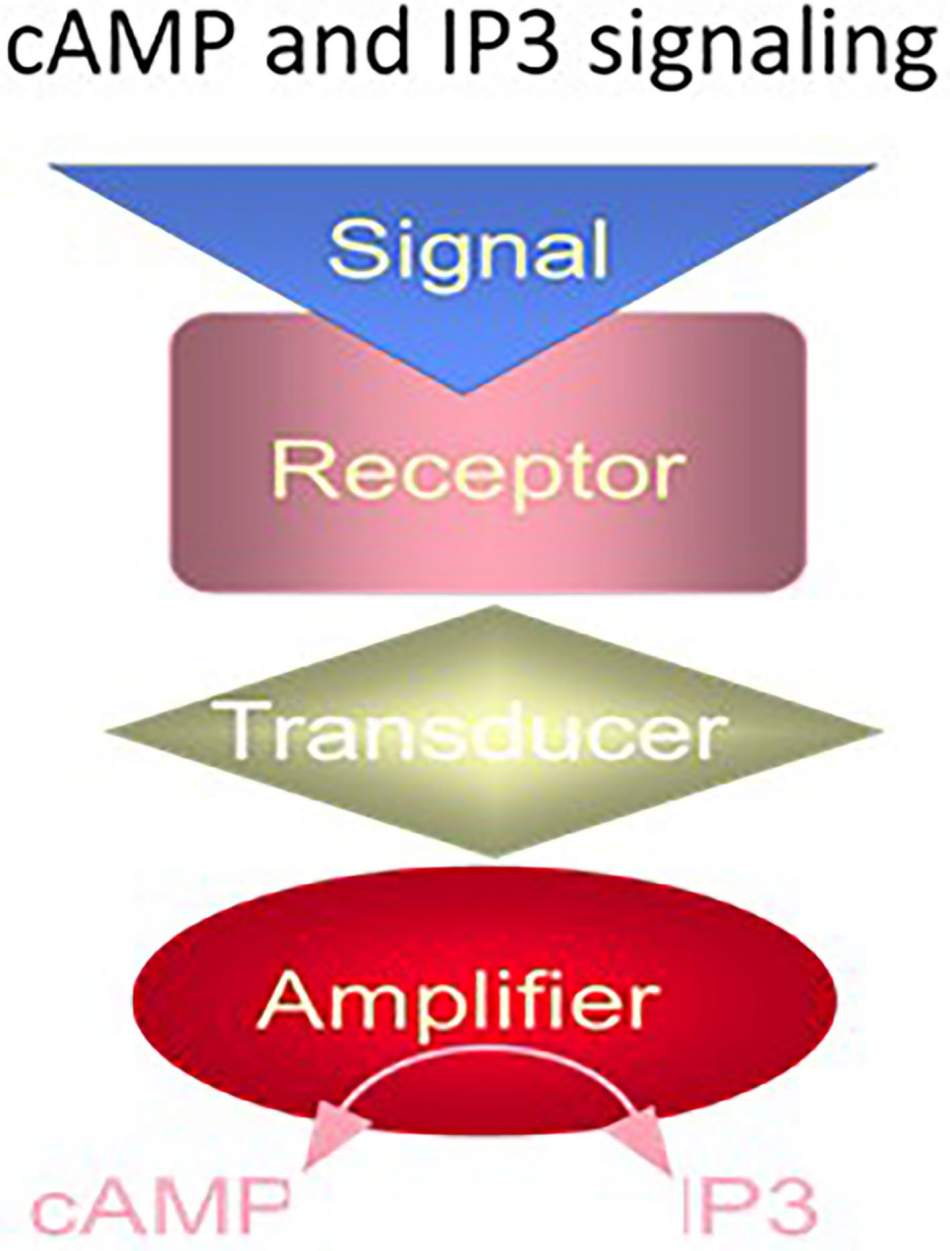
The cyclic AMP and IP3 signaling systems. The cyclic AMP and IP3 signaling systems consist of three separate components: hormone receptor, transducer (G protein) and amplifier adenylate cyclase (for cyclic AMP signaling) or phospholipase C (for IP3 signaling). (Modified from Sharma and Duda, 2014a).

The G proteins exist in two forms, stimulatory, G_s_, and inhibitory, G_i_; the former stimulates and the latter inhibits the transduction process. The molecular nature of the receptor specified the effect of the hormone. The cyclic AMP signaling system was thus a three-component system, composed of three distinct proteins – the receptor, the G protein, and the adenylate cyclase (Figure 1).

A similar G protein modulated pathway was discovered, subsequently. It was the phosphatidylinositol-signaling (reviewed in: Nishizuka, 1988; Berridge and Irvine, 1989; Bansal and Majerus, 1990; Harden, 1992; Strader et al., 1994). Here, the hormonal signal transformed phosphatidylinositol-4,5-bisphosphate (PIP_2_), a lipid signaling molecule into two separate second messengers, diacylglycerol and inositol triphosphate (IP_3_). IP_3_ signal mobilized the intracellular Ca^2+^ pool that could open TRP channels, activate protein kinase C or act on other Ca^2+^ sensors. Diacylglycerol also activated protein kinase C and certain TRP channels.

The two G protein cellular signaling pathways had a common structural feature, the hormonal receptor transducer was G protein. The receptor was a seven transmembrane-spanning protein. The hormone bound the receptor and generated cyclic AMP or IP_3_ *via* the amplifier component (Figure 1). The generated second messenger, cyclic AMP or IP_3_, through a cascade of biochemical reactions transduced the hormonal signal into a cell-specific physiological response.

Cyclic AMP signaling, modulated by the GTP-binding proteins, was the sole concept of cellular hormonal signaling from 1954 to 1970. Yet, relegated to its shadows, cyclic GMP had slowly begun to rise into prominence, and becoming an independent, third, hormonal signaling pathway. Its ascendance was, however, marked by un-surmounted hurdles by a few laboratories lead by Nobel laureates. Here, we briefly outline the sequential steps advancing the field of mammalian membrane guanylate cyclases, concentrating on their modular structures and complexity of their biochemistry and physiology. Note, that some contents are freely borrowed from earlier reviews (Sharma, 1978, 1985, 2002, 2010; Pugh et al., 1997; Sharma et al., 2004, 2014; Duda et al., 2005b, 2014; Sharma and Duda, 2012, 2014a,b).

## Hormonally-modulated MGC signal-transduction pathway

### Origin

Guanylate cyclase is an enzyme that catalyzes the transformation of GTP into cyclic GMP. Detection of cyclic GMP in rat urine (Ashman et al., 1963) and the observation that its concentration depended on the hormonal state (Hardman et al., 1969; Hardman and Sutherland, 1969) were the hints that it may be a hormonal second messenger. A model of membrane guanylate cyclase transduction system was first envisioned based on the cyclic AMP model. Finding of cyclic GMP and guanylate cyclase catalytic activity in the variety of tissues apparently supported this model (Goldberg et al., 1969, 1973; Ishikawa et al., 1969). It was short lived, however, with the superficial findings that the MGC activity was stimulated by various non-hormonal agents: polyunsaturated fatty acids, peroxides, hydroperoxides, free radicals, ascorbic acid, sodium nitroprusside, and even cigarette smoke (Goldberg and Haddox, 1977; Murad et al., 1979). Thus, the MGC lacked specificity and depended solely on cell’s oxidation–reduction potential. In radical outlooks, no component of cyclic GMP turnover was viewed as linked to an intracellular signaling. A case in point, because the known cyclic GMP-dependent protein kinase exhibited cross-reactivity with cyclic AMP-dependent protein kinase, cyclic GMP was deemed only as an enhancer for cyclic AMP signaling (Gill and McCune, 1979; reviewed in: Goldberg and Haddox, 1977; Murad et al., 1979). Thus, cyclic GMP was a sub-servant of the cyclic AMP signaling.

Despite this, a few groups, including ours, moved on to explore and then established a role for cyclic GMP as a second messenger in hormonal signal transduction (reviewed in Sharma, 1978, 1985; Sharma et al., 1988a,b; Pugh et al., 1997; Sharma and Duda, 2012, 2014a,b; Sharma, 2002, 2010; Duda et al., 2005b, 2014; Sharma et al., 1997, 2016).

Two critical cell models were instrumental in achieving this goal. One, an ACTH-sensitive MGC transduction system that solely exists in the rat adrenocortical carcinoma 494 cells and also in the isolated adrenal fasciculata cells (Perchellet and Sharma, 1980; Shanker and Sharma, 1980; Jaiswal and Sharma, 1986; Jaiswal et al., 1986; reviewed in Sharma, 2002, 2010). Two, unlike adrenal homogenates, isolated adrenal fasciculata and adrenocortical carcinoma cells lacked cyclic AMP phosphodiesterase activity. These were therefore ideal for settling whether cyclic AMP is the sole hormonal second messenger or whether ACTH evokes MGC activity (Kitabchi et al., 1971; Sharma, 1972). This issue has extensively been discussed in the most recent review (Sharma, 2022).

The conclusions were:

(1) “The ACTH-modulated biosynthetic step is the cleavage of the (20S)-20-hydroxycholesterol to corticosterone. This step is dependent on the generation of cyclic GMP, yet, not on the cyclic AMP. Accordingly, the step requires the labile synthesis of protein and is rate limiting” (Sharma, 1973; Sharma, 1974; Sharma and Brush, 1974).
(2) In the model of the carcinoma cells, steroidogenesis gets out of ACTH control, yet not of cyclic GMP.
(3) The physiological concentrations of ACTH, 2.5 to 10 μunits, do not increase the synthesis of cyclic AMP but stimulate the peak synthesis of cyclic GMP with a simultaneous rise in corticosterone synthesis.
(4) Thus, “the hormone, ACTH, modulates the steroidogenic production was supported by purification from the bovine adrenal cortex of the cyclic GMP-dependent protein kinase, the enzyme directly locked in with cyclic GMP” (Sharma et al., 1976; Ahrens and Sharma, 1979; Ahrens et al., 1982).
(5) The Mr. of the cyclic GMP-dependent protein kinase is 145,000. Two identical subunits (75,000 Da each) form the functional enzyme. The enzyme binds two molecules of cyclic GMP per holoenzyme and self phosphorylates. Calmodulin and troponin C, markedly stimulate the enzyme. The ability of cyclic GMP and its analogs to stimulate steroidogenesis matches their ability to activate cyclic GMP-dependent protein kinase. Thus, this protein kinase is totally different from the cyclic AMP-dependent protein kinase which dissociates into its regulatory and catalytic subunits upon binding cyclic AMP.

**Figure 2:**
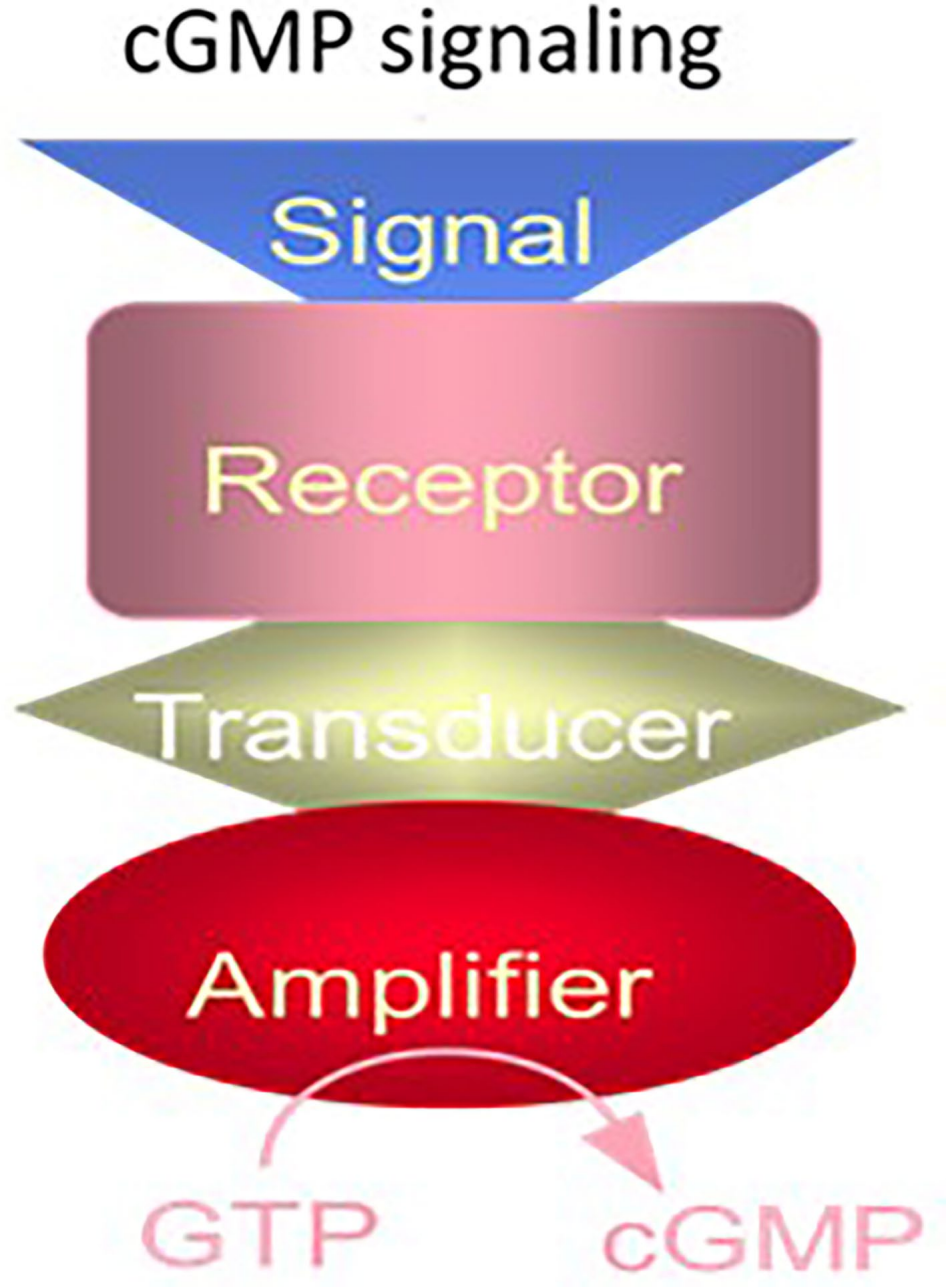
The cyclic GMP signaling system. The cyclic GMP signaling system consists of a single protein. The hormonal signal is recognized by the extracellular receptor domain; the signal is potentiated at the ATP-modulated ARM domain located next to the transmembrane domain in the intracellular portion of the protein (transducer) and the signal is amplified by the cyclase catalytic domain located at the C-terminus of the protein. (Modified from Sharma and Duda, 2014a).

These facts, and purification of the MGC from the rat adrenal cortex (Paul and Sharma, 1985), allowed us to propose the following model of MGC signaling (Figure 2).

While the studies on authenticity of the MGC signaling field were advancing, the investigators who originally doubted its existence took a turn around. Now, based on their new monoclonal antibody results with the lung soluble and MGC forms (Brandwein et al., 1981), they agreed on the independent existence of the MGC. Yet, they concluded, that biochemically and functionally the differences between both forms were only minor. Both forms were not the direct transducers of any hormonal signal. They responded only non-specifically to the common signals of hydroxyl radical, hydrogen peroxide, lipids and unsaturated fatty acids, oxidants, nitric oxide and a variety of other nitric oxide generating compounds such as nitrosoamines including cigarette smoke (Wallach and Pastan, 1976; Arnold et al., 1977; Mittal and Murad, 1977a; Murad et al., 1978a,b). This revised concept had two significant closings: (1) Underlying mechanism of action for all the nitrite-generating compounds is the same, *via* nitric oxide (NO) gas (Arnold et al., 1977; Katsuki et al., 1977; Mittal and Murad, 1977b; Waldman et al., 1982). (2) The NO activates the MGC at its catalytic site (Arnold et al., 1977).

They reinforced their concept by reporting the presence of NO-dependent MGC in almost all mammalian tissues, ranging from peripheral to the central nervous system. The response to NO varied, however; ranging from 3-fold stimulation in the liver to 14.7-fold in the rat cerebellum (Arnold et al., 1977).

### The extraordinary conclusion was that both forms of guanylate cyclases are modulated by No; and none is hormonally modulated

This passionate pursuit on the physiological irrelevance of the hormone-dependent MGC was extended to the cyclic GMP-dependent protein kinase. The belief was that it did not phosphorylate any protein with higher specificity than cyclic AMP-dependent protein kinase did. The implication was that in the instances where a stimulus (signal) generated cyclic GMP in the intact cell, it acted through a cyclic AMP transduction component instead of the cyclic GMP. Again, reinforcing the concept that the cyclic GMP signaling pathway was a subservient to the cyclic AMP transduction system (Gill and McCune, 1979).

Remarkably, for the discovery of the NO, and NO-modulated cyclic GMP signaling system, Robert Furchgott, Louis Ignarro and Ferid Murad were awarded the 1998 Nobel Prize in Physiology or Medicine. The underlying mechanism for the NO action was cited as: the NO gas generated in the endothelial cell layer, signaled guanylyl cyclase activation, and the generated cyclic GMP caused relaxation of the blood vessels.

Also, notably, in this concept, the soluble guanylate cyclase was the sole cellular signaling pathway.

The details for these awards, none-the-less, are misleading for two reasons. (1) It totally ignored the historical evolution of the MGC signal transduction. Factually, in the beginning these laboratories not only denied its cellular existence, they aggressively negated the ongoing results of the other laboratories that had established the physiological, biochemical and structural integrity of the membrane guanylate cyclase signal transduction. Before 1998, the date of the award, other groups, including ours in 1982, had already demonstrated that the guanylate cyclase exists in two forms, soluble and the MGC (Nambi et al., 1982). Only, the membrane bound form is locked with the hormonal signal transduction. And, this transduction system is not sub-servient to the cyclic AMP signaling system. (2) The awarded recipients believed that “both forms, membrane and soluble, guanylate cyclases were modulated by NO.”

The earliest documented facts, however, were that the membrane form is not NO-modulated and it is structurally and functionally different from the soluble form as summarized in Table 1 (according to Nambi et al., 1982).

**Table 1:** Summary of the properties of particulate and soluble guanylate cyclase of rat adrenocortical tissue (from Sharma, 2002).

**Conditions**	**Particulate**	**Soluble**
Abundance	80%	20%
ACTH	Stimulation	No effect
Sodium nitroprusside	No effect	Stimulation
Sodium azide	No effect	Stimulation
Tuftsin	No effect	Stimulation
Cd^2+^	Inhibition at high conc.	Inhibition at low conc.
	(EC_50_–400 μM)	(EC_50_–2 μM)
Dithiothreitol	No effect	Strong stimulation
N-Ethylamide	Inhibition	Stimulation at low and inhibition at high conc.

**Figure 3:**
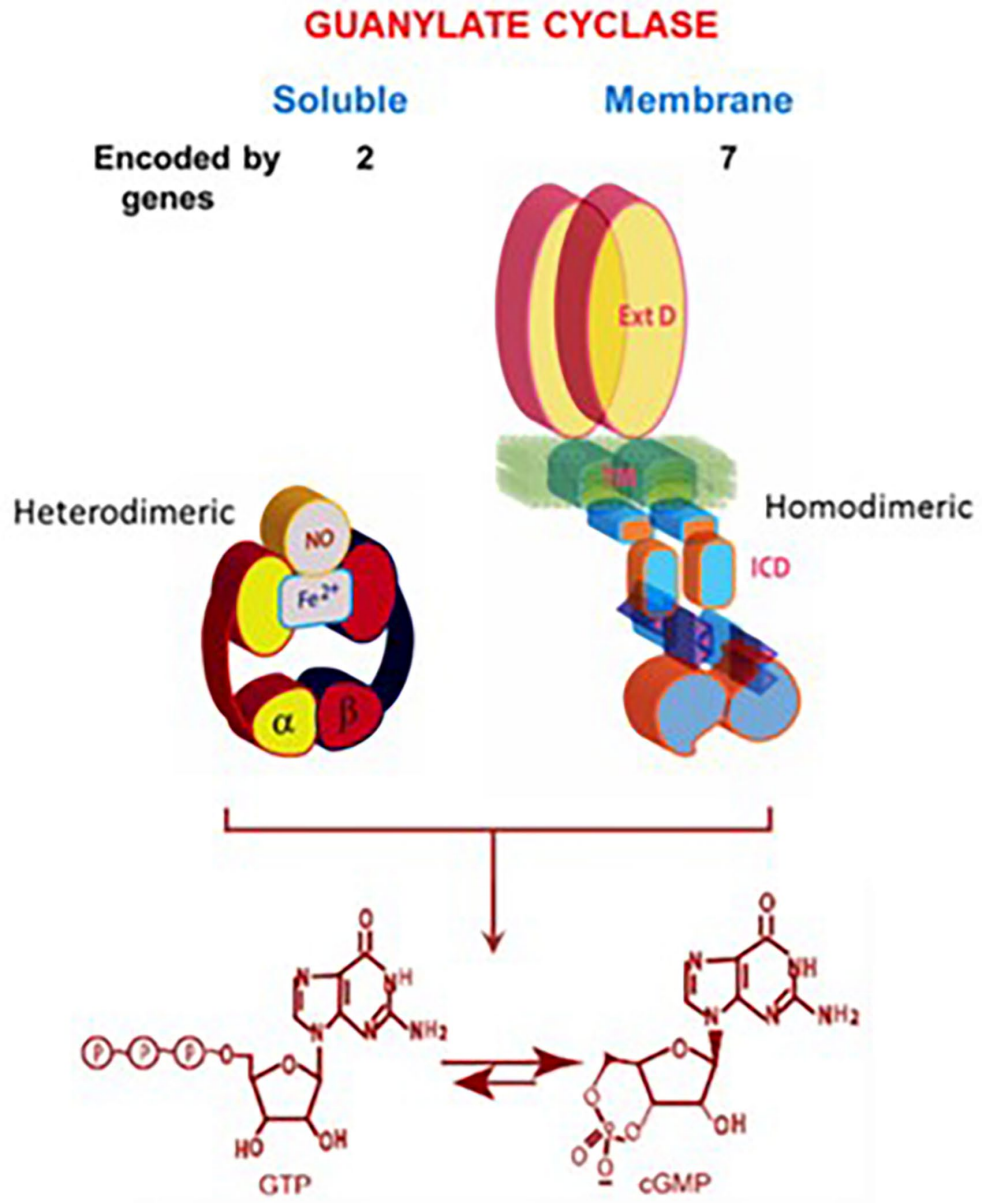
Soluble *vs*. membrane guanylate cyclase. Upper panel: Graphical representation of soluble and membrane guanylate cyclases. The soluble form is encoded by two genes; it is heterodimeric (subunits α and β) and requires heme for its activity. Both monomers contribute to the catalytic center, but their orientation is unknown yet (Allerston et al., 2013). The membrane guanylate cyclase is encoded by seven genes. It is a single transmembrane spanning protein. The extracellular domain (Ext) is located outside the cell; the transmembrane domain (TM) spans the plasma membrane; and the intracellular domain (ICD) is located inside the cell. The active form is homodimeric. The cyclase catalytic domain is located at the C-terminus of the protein. Both monomers in antiparallel orientation contribute to the catalytic center. Lower panel: Both cyclases are lyases (EC4.6.1.2) and catalyze synthesis of cyclic GMP from GTP. (From: (Sharma and Duda, 2014a).

The present understanding is that the soluble guanylate cyclase is modulated by NO, it is encoded by two genes (Denninger and Marletta, 1999), is heterodimeric and requires heme for its activity (Allerston et al., 2013). In contrast, the molecular structure of the membrane guanylate cyclase is totally different (Figure 3).

### Atrial natriuretic factor receptor guanylate cyclase (ANF-RGC) is the first purified and characterized MGC

The proposed structural model of the MGC purified from rat adrenocortical carcinoma (Paul and Sharma, 1985; Paul, 1986; Paul et al., 1987; Sharma et al., 1988a; Sharma and Duda, 2014b) and later from the rat adrenal cortex (Takayanagi et al., 1987a,b; Meloche et al., 1988) established that it is a receptor for atrial natriuretic factor (ANF); and, it is a three component transduction system, all components embodied in one protein. This MGC was named ANF-RGC, it specified the basis for ANF-stimulated cyclic GMP and corticosterone synthesis (Jaiswal et al., 1986). The narration in this original report is notable, “coexistence of the ANF receptor and guanylate cyclase activities on a single polypeptide chain indicates that the mechanism of transmembrane signal transduction involving mediation by second messenger, cyclic GMP, is different from the adenylate cyclase system. In hormone-dependent adenylate cyclase there is an assemblage of individual components – receptor, GTP binding protein, and catalytic moiety – for signal transduction (Figure 1). In contrast, the presence of dual activities – receptor binding and enzymatic – on a single polypeptide chain (Figure 2) indicates that this transmembrane protein contains both the information for signal recognition and its translation into a second messenger” (citation from Paul et al., 1987).

Thus, ANF-RGC was a multimodal signal transducer (Sharma et al., 1988a,b; Sharma, 2002). Its hallmark characteristics were that a hormone receptor domain projected extracellularly, a catalytic domain was inside the cell and a transmembrane domain linked the two. Its wide expression was shown through immunostaining by the antibody used to purify ANF-RGC to homogeneity from adrenocortical carcinoma and from adrenal cortex with neurons of the ventral horn region of rat spinal cord, cerebellar Purkinje cells, and renal glomerular cells (Ballermann et al., 1988; Marala and Sharma, 1988). A curious result of the early studies was that Ca^2+^ was needed for stimulation of steroidogenesis (Sayers et al., 1972; Haksar and Péron, 1973; Bowyer and Kitabchi, 1974), yet by itself, Ca^2+^ was ineffectual. Only with ACTH, did it become an effective steroidogenic factor (Perchellet and Sharma, 1979). Although the underlying mechanisms have not been known, Ca^2+^ would be, and was later found, to play a significant role in regulating MGC activity and multiplying its functionality.

In this manner the torturous debate questioning the independent existence of the hormone-dependent guanylate cyclase transduction system in mammalian systems ceased. Besides cyclic AMP and IP_3_, it was recognized as the third cellular signaling system. Extraordinarily, being independent of heme and NO gas modulation, it shattered the dogma that membrane guanylate cyclase signal transduction is modulated by these agents. This point is historically critical as narrated below.

In 1988, shortly after the Paul et al. (1987) paper was published (Paul et al., 1987), the Murad’s group, in a commentary in Science (Waldman et al., 1988) challenged our primacy in the discovery of ANF-RGC. The challenge was meritless, however (Sharma, 1988). By the authors’ own statement (Kuno et al., 1986), the enzyme, they purified from the rat lung, was not homogeneous but only 95% pure, leaving the possibility that the 5% contaminant contained the separate ligand binding or the cyclase catalytic activity. Also, in contrast to the 1:1 stoichiometry, the lung enzyme bound only 14.5% of ANF theoretical value and was stimulated by hemin, a characteristic of the soluble guanylate cyclase.

Significantly, these issues were never countered by the Murad’s group.

It was, thus, settled that ANF-RGC reported by our group was the first discovered surface receptor membrane guanylate cyclase. Particularly, it possesses two biological activities, a surface receptor and an enzyme. Subsequently, the ANF-RGC field grew logarithmically, with the new discoveries that (1) the source of ANF is the heart atria; (2) the heart is the endocrine gland; (3) ANF is the most potent hypotensive agent.

The studies initiated by the De Bold’s group and followed by others’ revolutionized the field of cardiovasculature and provided an opening for advancing the ANF research toward the development of drugs for hypertension (De Bold, 1982; Cantin and Genest, 1985; Schwartz et al., 1985; Atlas and Laragh, 1986; De Bold, 1986). It was shown that ANF regulates sodium excretion, water balance and blood pressure. And, significantly, ANF, a polypeptide hormone, besides ACTH, modulates the MGC activity. It, therefore, widened the mode of hormonal signal transduction providing clues for future explorations, basic and clinical.

With the knowledge that ANF-RGC is present in the adrenal gland, we asked two critical questions: (1) Does the heart-originated ANF signal modulate the steroidogenic machinery of the adrenal gland? And (2) how does ANF, *via* its surface receptor domain, activates the catalytic domain of ANF-RGC and generates cyclic GMP?

**Figure 4:**
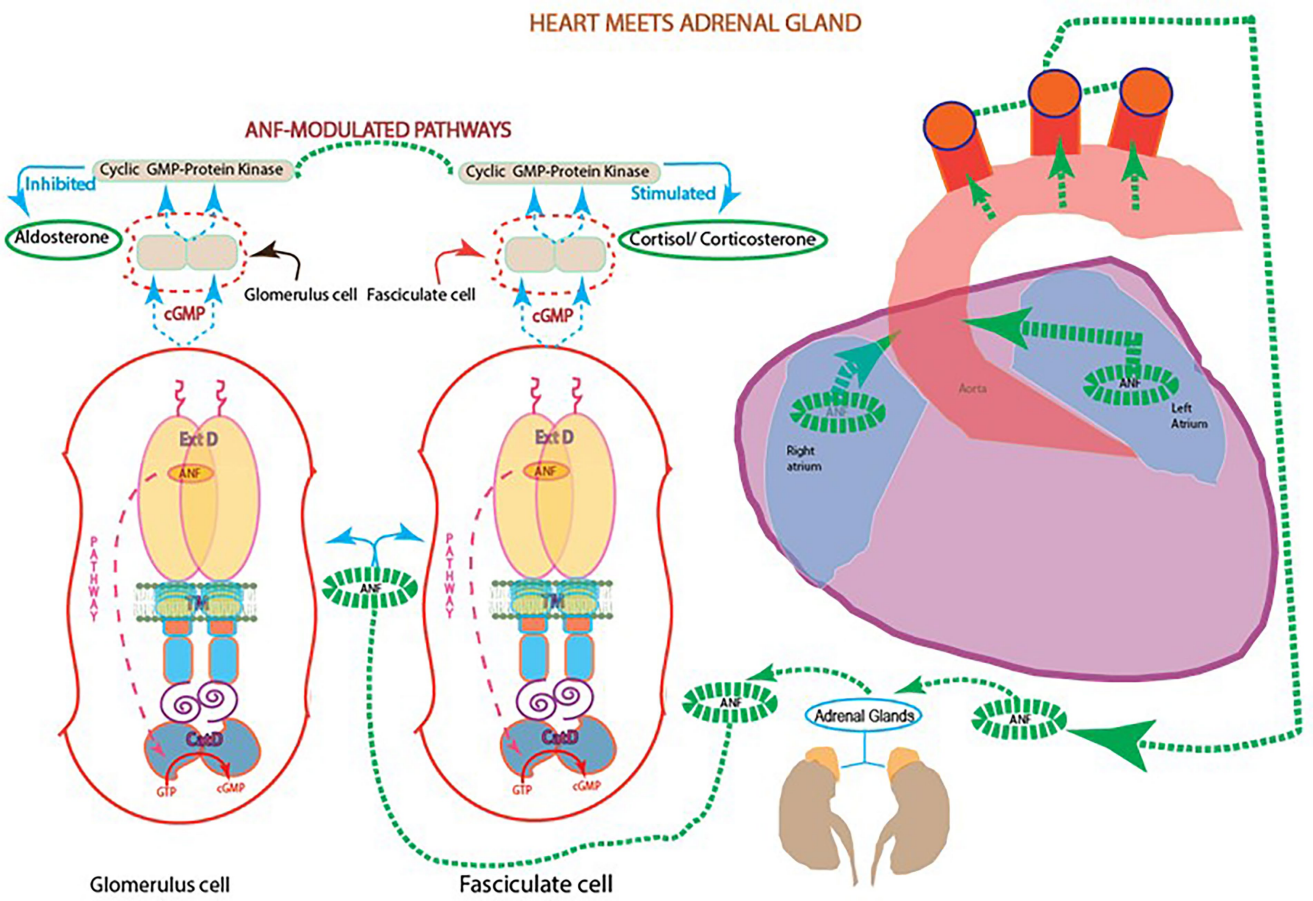
Heart meets adrenal gland: ANF reciprocal modulation of the two steroidogenic pathways. With every heartbeat, atria stretches and secretes ANF to the circulation. In the adrenal gland, ANF branches and initiates two signaling pathways involving cyclic GMP: one at the fasciculate cells to stimulate the production of cortisol/corticosterone; and the other at glomerular cells to inhibit the production of aldosterone and lower blood pressure.

In reference to (1), ANF-RGC presence in the glandular tissues of the adrenal gland and testis supported the idea that the heart signals the metabolic processes of steroidogenesis in these endocrine tissues. Accordingly, studies from various groups showed that ANF raises the levels of cyclic GMP and down-regulates the aldosterone formation in the adrenal glomerulosa cells (Atarashi et al., 1984; Chartier et al., 1984; De Léan et al., 1984; Kudo and Baird, 1984). Noting that aldosterone is the most potent hypertensive steroid hormone and ANF, the most potent hypotensive agent, the presence of ANF-RGC in the glandular tissues indicated that ANF-RGC is a SWITCH that links heart and these glands (Figure 4).

It was now possible to elucidate the molecular principles of this SWITCH and conquer the diseases of hyper-and hypo-tension. Indeed, as the future holds, this was one of the game changer contribution in the field of cardiology.

In hindsight, additional investigations started to flood in. They ranged from the purification of ANF-RGC from other glandular tissues to its cloning and its’ varied modes of steroidogenesis (reviewed in: Sharma, 2002).

Three years after the discovery of ANF-RC, its cDNA was cloned, and genetically tailored to probe the signaling site for ANF (Duda et al., 1991). Notably, purification of ANF-RGC expressed in COS cells using the antibody raised against the ANF-RGC purified from the rat adrenocortical carcinoma proved that the native and the cloned ANF-RGCs are immunologically identical.

Following the discovery of ANF-RGC, a very closely related MGC, type C natriuretic peptide receptor guanylate cyclase (CNP-RGC), was cloned first in a joint effort (Chinkers et al., 1989; Lowe et al., 1989) and then reported separately by the two laboratories (Chang et al., 1989; Schulz et al., 1989). The cloning of the mouse analog of ANF-RGC soon followed (Pandey and Singh, 1990). Another mRNA of a functionally defined form of MGC, STa-RGC, was cloned from the intestinal mucosa (Schulz et al., 1990; de Sauvage et al., 1991; Singh et al., 1991). This cyclase was a receptor for the bacterial enterotoxin and for the endogenous peptide hormones: guanylin and uroguanylin (Currie et al., 1992; Wiegand et al., 1992; Hamra et al., 1993; Khare et al., 1994).

Now, the identities of three forms of the MGCs were known. They, constituted a SURFACE RECEPTOR family. Two members, ANF-RGC and CNP-RGC were locked in with the physiology of cardiovasculature; third, STa-RGC, with the intestine. In a common theme, the family relaxed the vasculatures of their respective target organs and ANF-RGC also toned down the hypertensive activity.

### The receptor MGC transduction system is also present in the central nervous system

Prior to 1993 the consensus was that the surface receptor MGC signaling system existed exclusively in the periphery (Needleman et al., 1989; Brenner et al., 1990; Rosenzweig and Seidman, 1991). Yet, there were clues, that this may not be true. Ours and two other groups had documented the presence of ANF-RGC in the retina, through immunology and molecular cloning studies (Cooper et al., 1989; Duda et al., 1992; Kutty et al., 1992; Ahmad and Barnstable, 1993). Similarly, *via* the Northern-blot analysis presence of the CNP-RGC was detected in the central nervous system and in the cells of the neural crest (Kojima et al., 1990; Sudoh et al., 1990). Molecular cloning studies had established presence of CNP-RGC in the brain (Cooper et al., 1989). Yet, the attempts to demonstrate CNP-RGC in the retina had failed (Ahmad and Barnstable, 1993).

In 1993, our group entered the field of sensory neurons in the retina. The goal was to explore the presence of CNP-RGC there, and if confirmed, define its transduction system.

#### Cloning and expression

CNP-RGC cDNA was cloned from a phage human retina cDNA library (Duda et al., 1993b). The cDNA was then inserted into the pSVL vector and expressed in COS-7 cells. With the prior availability of the cloned pSVL-ANF-RGC, it was possible to compare the properties of these two MGCs.

#### MGCs embody a modular design

Hydropathy analysis showed that ANF-RGC, CNP-RGC and STa-RGC, are modular, with matching topographies. The extracellular domain, the receptor domain, binds a hormone and has maximal structural diversity. The intracellular portion begins with a tyrosine protein kinase-like domain and extends to a catalytic domain (Duda et al., 1991; PNAS). Using the recombinant tools and guided by the ANF-RGC structural template, it was possible to map the domains, and propose the mechanism of the transmembrane migration of the hormonal signal (reviewed in Sharma et al., 2016).

The first goal was to verify that the extracellular domain (ExtD) of ANF-RGC indeed houses the hormone’s (ANF) binding site. A GCα mutant of ANF-RGC, cloned from the adrenal cDNA library differed from ANF-RGC in two amino acid residues within the ExtD: position 338 was occupied by His instead of Gln and in position 364 Pro substituted for Leu (Duda et al., 1991). GCα exhibited cyclase catalytic activity but did not respond to ANF. Mutating the 338 and 364 residues of GCα to those of ANF-RGC restored ANF binding and ANF dependence in activation of the catalytic activity.

Thus, Gln^338^ and/or Leu^364^ are obligatory for ANF stimulation. Ensuing point mutation analyses singled out Leu^364^ as the key residue for both activities.

**Figure 5:**
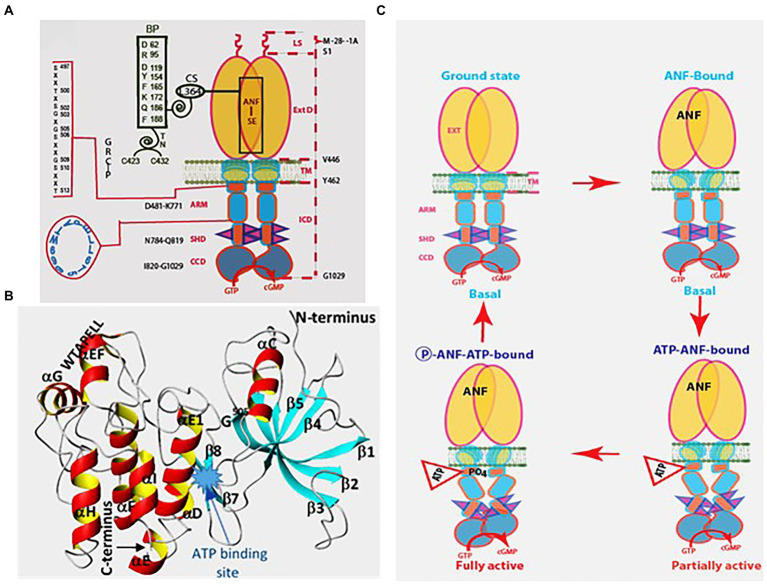
Model for signal transduction by atrial natriuretic factor-receptor guanylate cyclase (ANF-RGC). **(A)** Module segments involved in activation. An ANF-signaling element (ANF-SE) resides in the ExtD. A Central Switch (CS), L^364^, controls the ANF binding site. A binding pocket (BP) is hinged with the CS (van den Akker et al., 2000; Ogawa et al., 2004, 2009). Two disulfide bridged cysteine residues act as a transduction node (TN) to guide the transmembrane migration of the ANF signal to an intracellular, ATP-Regulated Module (ARM; Duda et al., 2005b). Significantly, the TN is active only in the hormone receptor guanylate cyclases but not in the photoreceptor guanylate cyclases (Shahu et al., 2022). ATP amplifies the ANF signal by bringing two critical domains to the surface: a glycine rich cluster G-X-G^505^-X-X-X-G, making surrounding serine and threonine available for phosphorylation (GRC-P) and a 7-aa residue W^669^-TAPELL^675^ motif to activate the core catalytic domain (CCD). **(B)** Structure of the ARM in its apoform. Four antiparallel β strands and one helix constitute the small lobe. The large lobe is made up of eight α helices and two β strands. The positions of the key G^505^ residue of the GRC motif within the small lobe and of the W^669^-TAPELL^675^ motif within the large lobe are indicated. ATP binding is sandwiched between the two lobes indicated by a star. **(C)** Activation model for ANF-RGC. Binding of an ANF molecule to the ExtDs of the dimer primes the ANF-SE, by rotating TN. The twisting motion propagates through TM to prepare ARM for ATP binding (Ogawa et al., 2004; Parat et al., 2010). ATP binding triggers a cascade of temporal and spatial changes (Duda et al., 2001c). With G^505^ in GRC-P acting as a pivot, the ATP binding site shifts its position and its floor rotates. There is movement of ARM’s β4 and β5 strands and the loop between them and movement of the αE and F helices that exposes the hydrophobic WTAPELL motif for interaction with CCD (Duda et al., 2009). These structural rearrangements initiate 50% maximal catalytic activity. Full activation is attained after multiple serines and threonines in GRC-P become phosphorylated (Duda et al., 2011b). The conformational changes wrought by ATP binding reduce the affinity of ANF-RGC for ANF and phosphorylation lowers the affinity for ATP binding. Dissociation of ANF and ATP return ANF-RGC to its ground state. (Modified from Duda et al., 2011b; Sharma et al., 2016).

Thus, the following features of ANF-RGC were proven: (1) ExtD, (2) the Leu^364^ of this domain is critically necessary for ANF binding, (3) in the intracellular domain the catalytic domain follows the kinase-like domain, and (4) the catalytic domain possesses both the basal and the hormone-dependent activities. Binding of ANF to the ExtD controls the catalytic activity in the intracellular domain (Figure 5A).

From the historic perspective, GCα, that was decisive in determining the ANF binding site and was the first mutant of any MGC linked with hormonally-dependent catalytic activity (Duda et al., 1991).

In CNP-RGC, Val^358^ corresponds to Gln^338^ and Glu^332^ was found to be vital for CNP binding and CNP-dependent cyclase activity; similar to Leu^364^ of ANF-RGC it does not have any effect on the basal cyclase activity of CNP-RGC (Duda et al., 1994). Replacing Gln^338^ residue with Glu bestows ANF-RGC with significant CNP-dependent activity (Duda et al., 1995).Therefore, Leu^364^ and Glu^332^ in ANF-RGC and CNP-RGC, respectively are equivalent in controlling the ligand specificities of these MGCs. Later, crystallographic analyses of the ExtD of ANF-RGC provided validation for the above and showed that this domain forms head-to-head homodimers (He et al., 2001; Ogawa et al., 2004, 2009).

### Hormonal signal transduction is ATP-regulated

A puzzling fact was that although purified ANF-RGC bound ANF stoichiometrically, its catalytic activity was unresponsive to ANF (Paul et al., 1987). This riddle was solved by two groups almost concomitantly, ours and Garbers’s, demonstrating that ATP is obligatory for ANF-dependent ANF-RGC activity (Chinkers et al., 1991; Marala et al., 1991). Neither ANF nor ATP alone can stimulate ANF-RGC activity. Because ATPγS and AMP-PNP, the non-hydrolyzable analogs of ATP, mirror ATP effect with EC_50s_ between 0.3–0.5 mM (Duda et al., 1993a), ATP must function as an allosteric regulator (Chinkers et al., 1991; Marala et al., 1991). The Hill coefficient for ATP binding was near 1 (Duda et al., 2011b), making it impossible to determine whether ANF-RGC binds one or two ATPs per dimer. Importantly, the same was found to be true for recombinant CNP-RGC (Duda et al., 1993c).

Molecular mechanism by which ATP controls transmission of the hormonal signal was elucidated through studies involving multiple techniques, site directed mutation, deletion and expression (reviewed in Sharma, 2010). They showed that the so-called “kinase homology domain, KHD” of ANF-RGC extends from a juxta-membrane domain (JMD) to the catalytic domain (Chinkers and Garbers, 1989; Marala et al., 1992). Within it, a glycine rich cluster (GRC), Gly^503^-Arg-Gly-Ser-Asn-Tyr-Gly^509^ confers ATP effect on ANF-dependent ANF-RGC activity (Goraczniak et al., 1992). This motif was, therefore, more appropriately named ATP-regulatory module (ARM; Figure 5A). The ARM in CNP-RGC is Leu^497^-Arg-Gly499-Ser-Ser-Tyr-Gly^503^. Replacing ANF-RGC’s ARM sequence with its counterpart in the CNP-RGC, has no effect on the ATP/ANF response (Duda et al., 1993a). These results were in agreement with an earlier finding that the KHDs of ANF-RGC and CNP-RGC are interchangeable (Koller et al., 1992) and that the transduction mechanisms of these two MGCs are identical. Mutational analyses of individual glycine residues in the ARM sequence pointed out to Gly^505^ and Gly^499^ in ANF-RGC and CNP-RGC, respectively, as essential for ATP binding and ANF signaling (Duda et al., 1993b). Thus, one residue controlled the both activities.

To determine the 3D-structure of the ANF-RGC ARM domain, homology-based modeling technique was used. The domain was modelled using the crystal structures of insulin receptor kinase and hematopoietic cell kinase as templates (Duda et al., 2000b; Sharma et al., 2001). Its functional determinants were deciphered and verified through point mutation/expression, time-resolved tryptophan fluorescence, Forster Resonance Energy Transfer, reconstitution and mass spectroscopic studies (reviewed in Sharma and Duda, 2014b).

The ARM consists of residues 481–771. It is composed of two lobes; a smaller, N-terminal of 91 residues (496–586) and larger of 185 residues C-terminal (587–771; Figure 5B; Duda et al., 2000b; Sharma et al., 2001). ATP binding results in repositioning of the W^669^-TAPELL^675^ motif within the larger lobe. This repositioning is obligatory for hormone-dependent activation of ANF-RGC; deletion of the WTAPELL motif caused unresponsiveness to ANF and ATP in the recombinant system (Duda et al., 2009).

In a genetically modified mouse model in which the WTAPELL motif was deleted from the ANF-RGC gene, hypertension and cardiac hypertrophy were observed (Duda et al., 2013; United States Patent No. 8835711 issued on September 16, 2014; Patent Publication Number: 20130291132). Thus, the seven-residue motif – WTAPELL – of ANF-RGC controls the hormonal regulation of blood pressure.

Binding of ATP causes that six buried serine and threonine of the smaller lobe are moved to the surface, and become available for phosphorylation by a hypothetical kinase. Phosphorylation is necessary for full ANF signaling. The maximal activation of ANF-RGC ceases upon ATP binding causing loss of the affinity for ANF (Larose et al., 1991; Jewett et al., 1993; Duda and Sharma, 1995a,b) and phosphorylation causes loss of the affinity for ATP; the enzyme undergoes homologous desensitization and returns to its basal state (Potter and Garbers, 1992). Based on these facts we proposed a working model for ANF-RGC activation (Figure 5C).

The same model was applicable to CNP-RGC and STa-RGC (Potter and Hunter, 1998; Bhandari et al., 2001; Jaleel et al., 2006). Later studies established that ATP regulation through the ARM is a unique feature of the hormonally-modulated subfamily of the membrane guanylate cyclases.

## Discovery of the Ca^2+^-modulated MGC subfamily locked in with phototransduction

SEEING, the beautiful gift of nature, starts with phototransduction, the conversion of light into an electrical signal in the outer segments of rod and cone photoreceptors. The historical finding of Koch and Stryer (1988) established that the retinal MGC is inhibited by [Ca^2+^]_i_ *via* a Ca^2+^ binding protein. Yet, the molecular nature of the MGC was not known and following the cyclic AMP model MGC was suspected to be a three separate components system – receptor, GTP-binding protein and adenylate cyclase – (Stryer, 1986).

From 1988 to 1991, search for the molecular identity of the vision-linked MGC was actively pursued. Yet, the conclusions were confusing and often flawed (reviewed in Pugh and Cobbs, 1986; Pugh et al., 1997).

Finally, four independent laboratories reported purification of the retinal MGC. Its biochemical characteristics reported by three were similar (Hayashi and Yamazaki, 1991; Koch, 1991; Margulis et al., 1993). The molecular mass of MGC was 110–120 kDa and importantly, its catalytic activity was unresponsive to ANF and to the [Ca^2+^]_i_ alone.

In sharp contrast, the fourth laboratory reported its Mr. of 67 kDa. And, notably the MGC was nitric oxide-dependent (Horio and Murad, 1991a,b). Later results, including from our group, demonstrated that these conclusions were flawed. This MGC was neither a 67 kDa protein, nor its catalytic activity was NO-modulated.

For historical reasons these studies from our group are briefly narrated below. For the first time they provided unequivocal identity of the wild type MGC at the biochemical and the molecular level. Reconstitution studies showed that ROS-GC was [Ca^2+^]_i_ modulated with a pattern that mirrored the physiological conditions of phototransduction.

The MGC was purified from the ROD OUTER SEGMENTS of the bovine retina (Margulis et al., 1993). Hence named ROS-GC. Based on the protein sequence of it four fragments, its cDNA was cloned from the bovine retina cDNA library (Goraczniak et al., 1994). The cloned enzyme was unresponsive to ANF and CNP and showed an overall minimal, 27%–30%, sequence identity with the three hormone receptor guanylate cyclases – ANF-RGC, CNP-RGC and STa-RGC (Goraczniak et al., 1994).

Comparison of the biochemical estimates with those acquired from cloning/expression experiments led to the realization that ROS-GC aminoacid sequence includes an N-terminal leader sequence (LS) that gets deleted post-translationally. The calculated molecular mass of ROS-GC with the 56-amino acid LS is 120,361 Da; without it, it is 114,360 Da (Goraczniak et al., 1994). The 114,360 Da molecular mass was quite close to the previously determined bovine (Koch, 1991) and toad photoreceptor guanylate cyclases (Hayashi and Yamazaki, 1991). A second ROS-GC, ROS-GC2, was discovered in bovine retina (Goraczniak et al., 1997) shortly thereafter and its human variety was termed Ret-GC2 (Lowe et al., 1995). Theoretically, ROS-GC1 and ROS-GC2 could organize into homodimers and heterodimers, but in reality only few, if any, heterodimers are formed in the retina despite the co-expression of both guanylate cyclases in rods (Yang and Garbers, 1997). As of today, no extracellular ligand has been identified for ROS-GC therefore, it remains an orphan receptor.

Proper identification of ROS-GC had an important historical impact. It corrected an earlier structural flaw.

In 1992 Shyjan et al. reported the molecular cloning of a membrane guanylate cyclase from the human retina (Shyjan et al., 1992) and named it retGC. Because retGC *via in situ hybridization* analysis was detected in the inner segments and outer nuclear layers of the monkey’s retina and the cyclase was different from ANF-RGC and CNP-RGC, the authors suggested that it may be a part of the phototransduction machinery (Shyjan et al., 1992).

The cloning and identification of ROS-GC proved, however, that retGC was not ROS-GC. There were critical structural differences between them. Remarkably, in 1995, the structure of retGC was revised (Lowe DG, accession number M92432), yet not published, to show its complete identity with the structure of ROS-GC, published in the early part of 1994 (Goraczniak et al., 1994). Thus, ROS-GC structure helped to establish retGC as the human counter part of bovine ROS-GC.

The discovery of mutations in ROS-GC1 linked with retinal disorders made it possible to establish the critical role of ROS-GC1 in phototransduction and define its abnormalities in molecular terms (Duda et al., 1999a,b, 2000a; Tucker et al., 1999; Wilkie et al., 2000; Ramamurthy et al., 2001).

### The modulation of ROS-GC occurs *via* Ca^2+^-binding proteins

Long before the molecular identity of the first Ca^2+^-modulated ROS-GC1 was known, Koch and Stryer provided the evidence that a calcium binding protein stimulates MGC activity in ROS in a [Ca^2+^]-dependent fashion (Koch and Stryer, 1988). Subsequently, two Ca^2+^-binding proteins were purified and cloned from the retina (Palczewski et al., 1994; Dizhoor et al., 1995; Frins et al., 1996). They were termed GCAP1 and GCAP2, and have been linked with phototransduction.

A third GCAP, GCAP3, was also cloned from the retina (Haeseleer et al., 1999). It appears to be cone-specific but the molecular mechanisms underlying its function remain largely unknown.

**Figure 6:**
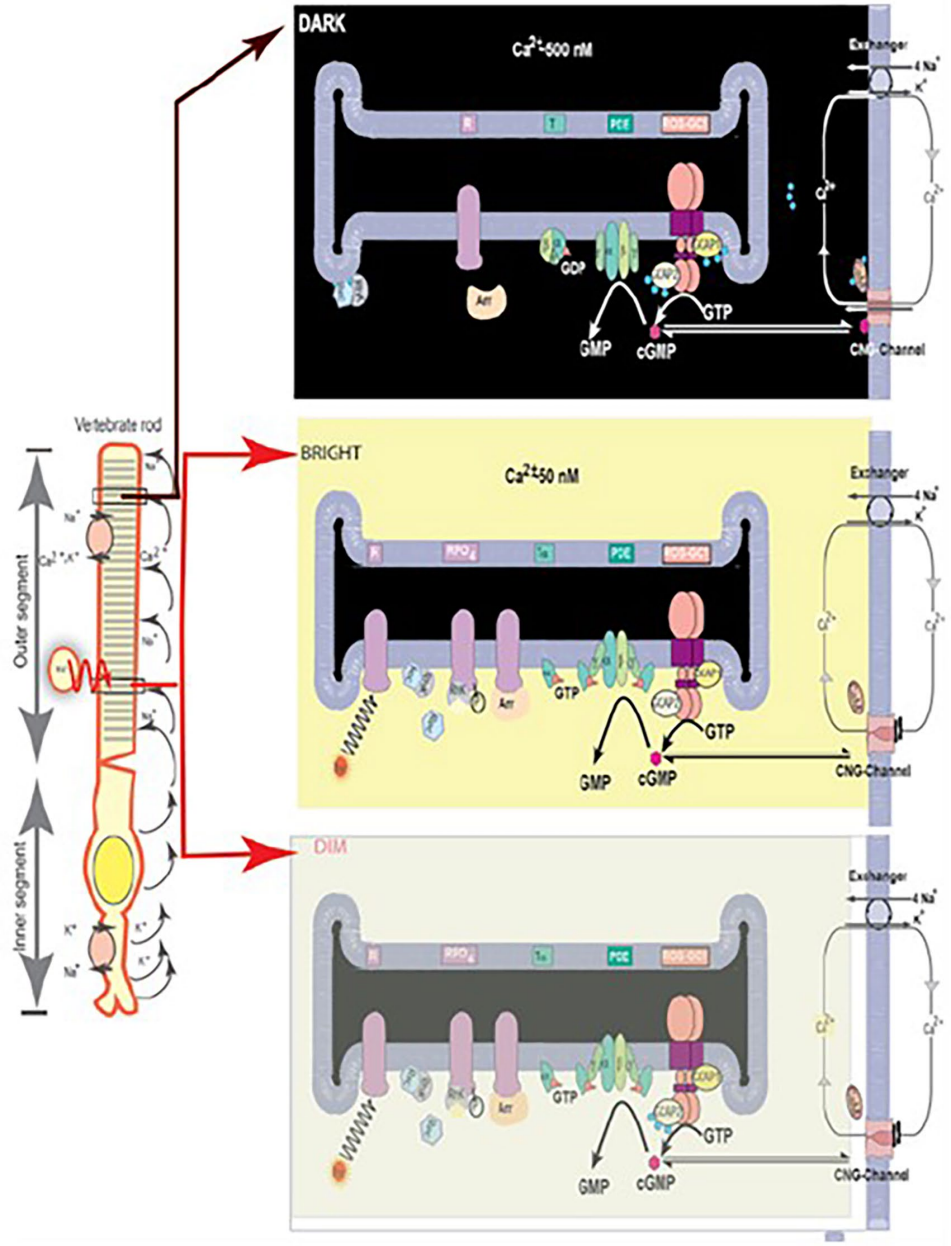
Schematic representation of the luminosity-dependent operation of the ROS-GC-GCAP transduction system. Left panel. An illustration of a typical vertebrate rod. In the DARK a circulating current (arrows) is present. It is outward in the inner segment and carried primarily by K^+^; in the outer segment the net charge is inward, with about 90% of the inward flow carried by the Na^+^ and 10% by Ca^2+^ ions. Na^+^/K^+^ exchange pumps in the inner segment membrane and Na^+^/K^+^-Ca^2+^ exchangers in the outer segment membrane (see also right panels) maintain the overall ionic gradients against the dark flows. The capture of a photon (hν) by a rhodopsin molecule in one of the disc membranes of the outer segment initiates the photo-transduction cascade. Right upper panel, DARK. The components of the Photo-Transduction cascade are shown in the dark/resting steady-state. Cytoplasmic cyclic GMP (red circle), generated by the basal catalytic activity of ROS-GC, keeps a fraction of CNG channels in the plasma membrane open. ROS-GC1 *via* its ^415^M-L^456^ segment is GCAP1-and *via* ^965^Y-N^981^ is GCAP2-bound. Ca^2+^ ions enter the cell *via* the CNG-channel and are extruded *via* the Na^+^/K^+^, Ca^2+^-exchanger. Synthesis and hydrolysis of cyclic GMP by ROS-GC and PDE, respectively, occur at a low rate. The heterotrimeric G protein, transducin, is in its GDP-bound state and is inactive. The Ca^2+^ binding proteins calmodulin (CaM), recoverin (Rec) are bound to their target proteins, the CNG-channel, rhodopsin kinase (Rhk), respectively. Right middle panel. Absorption of BRIGHT LIGHT by the visual pigment rhodopsin leads to the activation of the transduction cascade: the GTP-bound α-subunit of transducing activates PDE that rapidly hydrolyzes cyclic GMP. Subsequently, the CNG-channels close and the Ca^2+^-concentration falls. The fall in cytoplasmic [Ca^2+^]i is sensed by Ca^2+^-binding proteins: CaM dissociates from the CNG-channel what leads to an increase in cyclic GMP sensitivity of the channel; recoverin stops inhibiting rhodopsin kinase; rhodopsin becomes phosphorylated. Both Ca^2+^-free GCAPs in their changed configurations activate ROS-GC and synthesis of cyclic GMP increases. Arrestin (Arr) binds to phosphorylated rhodopsin and interferes with the binding and further activation of transducin. Enhancement of cyclic GMP synthesis brings it to its original DARK state level and termination of the cascade, which leads to reopening of CNG channels. Right bottom panel, DIM LIGHT. The initial fall of [Ca^2+^]_i_ is selectively detected only by GCAP1. In its Ca^2+^-free state GCAP1attains the activated mode and stimulates ROS-GC activity. GCAP2 remains Ca^2+^-bound and in its inhibitory mode. (Reproduced from Sharma and Duda, 2014a).

The phototransduction model presented in Figure 6 shows that ROS-GC in its native state is bound to GCAPs. Fluctuating levels of [Ca^2+^]_i_ change the conformation of GCAP/s. This, in turn, regulates the cyclase through specified modules in ROS-GC. These model features have been borrowed from (Sharma and Duda, 2014a) and they incorporate our and others conclusions (Pugh et al., 1999; Burns and Baylor, 2001; Koch et al., 2002; Sharma, 2002; Koch, 2006; Luo et al., 2008; Stephen et al., 2008; Wensel, 2008; Koch et al., 2010; reviewed in Koch et al., 2010; Sharma, 2010; Koch and Dell’Orco, 2013). Notably, the model also explains the results of the GCAPs null mice study that questioned the role of GCAP2 in phototransduction (Howes et al., 2002).

**Figure 7:**
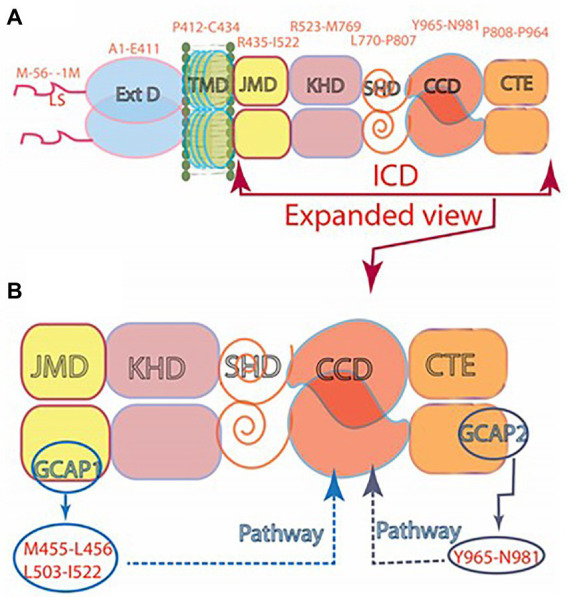
**(A)** Modular construction of the ROS-GC1 dimer. A 56 aminoacid leader sequence (LS) precedes the extracellular domain (ExtD) in the nascent, immature protein. All signaling events occur in the intracellular domain (ICD), which is composed of: JMD, juxtamembrane domain; KHD, kinase homology domain; SHD, signaling helix domain; CCD, catalytic core domain; and CTE, C-terminal extension. **(B)** Interaction with GCAP1 and GCAP2. Two specific switches for Ca^2+^ sensing subunits, one for GCAP1 in the JMD, and one for GCAP2 in the CTE, are located on opposing sides of the CCD. The MGC complex exists as a dimer of homodimers in which two ROS-GC1s combine either two GCAP1s or two GCAP2s.

It was thereby established that (1) Vision begins with PHOTOTRANSDUCTION, i.e., the conversion of light (photon) into an electrical signal in the photoreceptors outer segments. (2) Ca^2+^ and cyclic GMP are essential components of the photoreceptors’ response to a captured photon. (3) Significantly, cyclic GMP is the second messenger of phototransduction; it increases the conductance of ion channels (Fesenko et al., 1985). (4) ROS-GC (1 and 2) is the source of the cyclic GMP. (5) ROS-GC1 *in statu nascendi* contains N-terminal leader sequence (LS) that is deleted post-translationally; the calculated molecular mass of the protein with the LS is 120,361 Da; without it, 114,360 Da. (5) No extracellular modulator was found for ROS-GC. (6) Ca^2+^-free GCAP1 activates ROS-GC1 but Ca^2+^-bound (IC_50_ ~ 100 nM), becomes an inhibitor (Dizhoor and Hurley, 1996; Duda et al., 1996b). Thereby, at low and high Ca^2+^ levels, GCAP1 is a Ca^2+^-sensing subunit of the ROS-GC1. (7) In recombinant system, GCAP2 is a more powerful stimulator of ROS-GC1 activity at low Ca^2+^ but with significantly higher than for GCAP1 EC_50_ (compare of 6–8 mM for GCAP2 and 0.8 mM for GCAP1; Duda et al., 1996b; Goraczniak et al., 1998; Krishnan et al., 1998). (8) Ca^2+^-free GCAP2 with an EC_50_ of 1 mM stimulates ROS-GC2 activity by 12-fold whereas GCAP1 is unable to do so (Duda et al., 1996b; Goraczniak et al., 1998); thus, GCAP2 is the only modulator of ROS-GC2 activity. (9) ROS-GC1 binds the two GCAPs are different sites; GCAP1 at M^445^-L^456^ and L^503^-I^522^ (Lange et al., 1999); GCAP2 at Y^965^-N^981^ (Duda et al., 2005a; Rehkamp et al., 2018; Figure 7).

In contrast, one group proposes that both GCAPs bind to the same site on ROS-GC1 (Peshenko et al., 2015a,b).

(10) Even though GCAPs bind to opposite sides of the ROS-GC1’s CCD, they both use the W^657^-TAPELL^663^ motif for stimulation of the cyclase catalytic activity at low Ca^2+^ (Duda et al., 2011a). Finally, (11) studies on double knockout mice demonstrated that without ROS-GC1/ROS-GC2, rods and cones do not respond to light. Expression of a third guanylate cyclase and a third GCAP linked with phototransduction is therefore excluded in mouse.

A third GCAP, GCAP3, is present in the human retina and in reconstitution system it stimulates ROS-GC1/ROS-GC2 in a Ca^2+^-dependent manner similar to that of GCAP1. Its role in phototransduction is unclear, however (Haeseleer et al., 1999). Most recent study suggests that mutation in GCAP3 may lead to retinitis pigmentosa (Avesani et al., 2022). Apparently mammalian photoreceptors utilize a Ca^2+^-modulated system composed of a pair of ROS-GCs and up to three GCAPs in their outer segments. Studies on mice deficient for either GCAP1 or GCAP2 support these conclusions (Makino et al., 2008, 2012; Koch and Dell’Orco, 2013). GCAP1 has lower affinity for Ca^2+^ and is the first to sense light-induced fall in intracellular Ca^2+^ and start activating ROS-GC. Over time after, when Ca^2+^ concentrations are further lowered the effect of GCAP1 saturates and GCAP2 takes over stimulation of ROS-GC activity. Together, the two GCAPs limit the growth of the photon response and accelerate the kinetics of the response recovery (Mendez et al., 2001 and Figures 4A,B in Sharma et al., 2016).

Although ATP is not required, yet it raises the ROS-GC1 basal catalytic activity and GCAP1-stimulated activity at low Ca^2+^ (Gorczyca et al., 1994; Aparicio and Applebury, 1996). ROS-GC1 exhibits self-phosphorylating kinase activity. Four serine residues in its ARM are subject to phosphorylation due to this activity (Aparicio and Applebury, 1996; Bereta et al., 2010) yet, its purpose remains unknown; it does not affect the basal and GCAP1-stimulated activities. Additional accounts of properties of ROS-GCs in control of phototransduction can be found in (Sharma and Duda, 2014a; Wen et al., 2014; Koch and Dell’Orco, 2015).

The discovery that the synthesis of cyclic GMP in photoreceptor outer segments is Ca^2+^-dependent added another dimension to the understanding of MGC signal transduction. It is not limited to the transduction of hormones and other extracellular signals but is able to sense intracellular signals as well.

### Recoverin, a misplaced molecule that diverted the MGC research

In an enormous historical prospective, the progression of the phototransduction field will leave behind a huge voyage if the story of RECOVERIN is not narrated.

After the seminal observation that a soluble fraction of bovine ROS causes activation of the photoreceptor MGC in the absence of [Ca^2+^] (Koch and Stryer, 1988), the structure of ROS-GC was believed to be composed of “separate regulatory and catalytic subunits” (Stryer, 1991). In hindsight, as indicated earlier, this belief was powered by the influence of the cyclic AMP signaling pathway involving three separate subcomponents – hormone surface receptor, GTP-binding protein and catalytic (adenylate cyclase; Figure 1). With the unknown structure of ROS-GC and its modulation, the groups from the United States and the former Soviet Union joined forces to decode the molecular principles of a protein that appeared to regulate guanylate cyclase activity (Dizhoor et al., 1991) and adhered to the functional description of the protein described by Koch and Stryer (1988). They named it RECOVERIN “because it promoted recovery of the dark state” of phototransduction. For 2 years this concept remained dominant.

However, the purer preparations of recoverin showed a disturbing decline in guanylate cyclase stimulation. It became evident that recoverin was not the sought after regulator of guanylate cyclase activity. Thus, the initial conclusion was withdrawn (Hurley et al., 1993).

Later findings demonstrated that recoverin had no role in the ROS-GC modulation. Rather it exerted Ca^2+^-dependent control over the phosphorylation of photo-excited rhodopsin by rhodopsin kinase (Kawamura, 1993; Chen et al., 1995; Klenchin et al., 1995).

At almost the same time, GCAP1 and GCAP2 were discovered, and it became evident that they indeed stimulate ROS-GC catalytic activity (Palczewski et al., 1994; Subbaraya et al., 1994; Dizhoor et al., 1995; Gorczyca et al., 1995; Frins et al., 1996). Thereby, the saga of RECVERIN in the modulation of ROS-GC ended.

### ROS-GC is a bimodal Ca^2+^ switch

With the discovery of GCAPs the dogma developed that they are the sole modulators of ROS-GC activity. The curious aspect was, however, that the GCAPs/[Ca^2+^]_i_ inhibited ROS-GC activity, yet, in surface receptor MGC family, the ligand, stimulated the MGC activity. The following questions were explored: (1) Do the vision linked retinal neurons embody an additional ROS-GC linked pathway where [Ca^2+^]_i_ stimulates it? (2) If, “yes,” how does it operate?

The answer to the first question was, yes. To the second, that the ligand of ROS-GC1was S100B protein (*vide infra*).

When the studies on GCAPs regulation of ROS-GC activity were in full swing, our group purified another Ca^2+^-dependent regulator of ROS-GC’s activity from the retina; it was named Ca^2+^-dependent GCAP (CD-GCAP; reviewed in Sharma et al., 2014). CD-GCAP turned out to be an isomer of the brain S100B (the only commercially available form; Pozdnyakov et al., 1995, 1997; Duda et al., 1996a, 2002; Margulis et al., 1996; Wen et al., 2012). The retinal form has Ca^2+^-bound whereas the brain form is Zn^2+^-bound. It is of importance because the cations bound result in opposite effects of S100B on ROS-GC activity; the Zn^2+^-bound form inhibits while the Ca^2+^-bound form stimulates ROS-GC1 (Pozdnyakov et al., 1997). Here, we focus on the retinal form.

The evidence that S100B directly interacts with ROS-GC1 came from cross-linking experiments. Biochemical parameters of this interaction were determined through surface plasmon resonance spectroscopy. S100B binds ROS-GC1 with a K_1/2_ of 198–395 nM (Duda et al., 2002) and stimulates cyclic GMP synthesis with a K_1/2_ for Ca^2+^ of ~400 nM (Duda et al., 1996a). Peptide competition experiments pointed out to two C-terminal segments: aa 962–981 and 1,030–1,042 on ROS-GC1 as the binding sites for S100B. This was confirmed by the deletion mutagenesis/expression experiments. They narrowed down to R^966^-IHVNS^972^ motif as the binding site and the R^1039^–RQK^1042^ flanking cluster as promoting maximal ROS-GC1 activation (Duda et al., 2002). S100B is a small protein with molecular weight of 10 kDa; it has only 2 EF Ca^2+^ binding hands, and organizes into a tetramer (Donaldson et al., 1995).

Since in the recombinant reconstituted systems ROS-GC1 has the ability to interact with the GCAPs and S100B, it could operate as a bimodal Ca^2+^ switch under the conditions that a cell expresses all three components. The possibility was tested using isolated photoreceptor-bipolar synaptic membranes of the bovine retina. This region was chosen because prior immunohistochemical studies had demonstrated the co-presence of ROS-GC1 with GCAP1 and S100B there (Liu et al., 1994; Cooper et al., 1995; Duda et al., 2002). Indeed, these membranes exhibited high ROS-GC1 activity at 10 nM [Ca^2+^]_i_, the activity decreased as [Ca^2+^]_i_ raised to a few hundred nM and then, when Ca^2+^ concentration was about 1 mM, it raised again (Figure 5 in Venkataraman et al., 2003).

The presence of ROS-GC1, GCAPs and S100B in the photoreceptor outer segments (Cuenca et al., 1998; Kachi et al., 1999; Rambotti et al., 1999) implicated a role for bimodal switching in phototransduction. The possibility was probed with mouse knockout models: S100B^−^/^−^, GCAP1^−^/^−^, GCAP2^−^/^−^, GCAP1/GCAP2^−^/^−^, ROS-GC1^−^/^−^. Biochemical experiments demonstrated functional linkage of S100B with ROS-GC1 but not with ROS-GC2 at [Ca^2+^]_i_ > 200 nM in the generation of cyclic GMP. Although these concentrations exceed the physiological range for Ca^2+^ in mouse ROS (Woodruff et al., 2002), they were appropriate for cones, which sustain higher levels of Ca^2+^ in darkness. It was therefore reasonable that the most recent immunocytochemistry co-localizes S100B and GCAP1 with ROS-GC1 in murine retinal cone outer segments but not ROS (Wen et al., 2012).

### A new Ca^2+^ sensor neurocalcin δ (NCδ) is expressed in the retinal neurons and modulates ROS-GC activity

With the finding that ROS-GC signaling pathway in the retinal neurons is governed by the Ca^2+^-sensor proteins, GCAPs, and S100B, the possibility was explored for the existence of other similar pathway/s in these neurons. One such pathway was found. It was modulated by the Ca^2+^-sensor NCδ (comprehensively reviewed in Sharma et al., 2016).

Found in the neurons of the inner retina, the sequence of NCδ is 35% identical with that of GCAPs. Purified from the bovine brain, Ca^2+^-free NCδ has no effect on recombinant ROS-GC1 activity. Yet, when Ca^2+^-bound, it stimulates the MGC catalytic activity with a K_1/2_ for Ca^2+^ of 0.8 mM (Kumar et al., 1999; Krishnan et al., 2004). NCδ stimulates only ROS-GC1; it remains ineffective with ROS-GC2. Functionally, NCδ is comparable to S100B but not with the GCAPs. One molecule of NCδ has four EF Ca^2+^-binding hands (as GCAPs have) while S100B has only two. Despite significant sequence differences between S100B and the C-terminal half of NCδ (GenBank accession numbers NP^_^001029727.1 and NP^_^776823.1, respectively), their Ca^2+^-bound crystal structures are very similar with respect to helix-packing arrangements of their EF hands (Vijay-Kumar and Kumar, 1999). Thus, NCδ and S100B are structural and functional analogs.

NCδ is a homodimer of 20 kDa (Krishnan et al., 2004; Venkataraman et al., 2008). Similar to other NCSs, it exhibits a Ca^2+^-dependent electrophoretic mobility shift (Ladant, 1995; Frins et al., 1996). Out of its four EF-hand motifs, only three – EF2, EF3, EF4 – are functional in binding Ca^2+^ (Okazaki et al., 1992; Terasawa et al., 1992; Vijay-Kumar and Kumar, 1999). Following the NCS family trait, NCδ is myristoylated at the N-terminus; the only known exception from the trait is the Kv-channel interacting protein subfamily (Braunewell and Gundelfinger, 1999; Burgoyne and Weiss, 2001). Ca^2+^ myristoyl switch determines NCδ activity. In the absence of Ca^2+^ myristoyl group is buried within NCδ hydrophobic pocket but becomes exposed upon Ca^2+^ binding enabling membrane binding of the protein (Lim et al., 2011). From the functional viewpoint, NCδ is a true subunit of ROS-GC1 transduction complex; because the resting intracellular Ca^2+^ concentration of 100–200 nM is sufficient to keep some of its molecules associated with ROS-GC1 thus, allowing very short response time. NCδ and ROS-GC1 are co-present together in the inner plexiform layer of the retina where addition of Ca^2+^ stimulates ROS-GC1 activity (Krishnan et al., 2004); the myristoylation of NCδ causes a 2-fold amplification of the ROS-GC1activity. Determined by surface plasmon resonance spectroscopy, Ca^2+^-bound NCδ attaches ROS-GC1 with *K*_A_ = 2.3 × 10^6^ M^−1^ and *K_D_* = 4.6 × 10^−7^ M (Krishnan et al., 2004).

Mapping studies defined that the NCδ binding site resides within the aa732–962 stretch of ROS-GC1 (Krishnan et al., 2004). The site does not overlap with the GCAP1, GCAP2-, or S100B-binding domains of ROS-GC1 (*vide supra*). Finer analyses pin pointed the modulated site to the V^836^-L^857^ region located in the heart of the ROS-GC1 catalytic domain (Venkataraman et al., 2008; note: the numbering in this reference is offset by one aminoacid). Spatially, this segment of ROS-GC1 accommodates the V-shaped crevice of NCδ, and, it forms a distinct hydrophobic–hydrophilic patch, a characteristic feature of the Ca^2+^-dependent signaling property of NCδ. It has a secondary structure of the helix–loop–helix. It is acidic with a pI of 3.37 (Venkataraman et al., 2008).

These findings challenged the existing concept that the CCD, located at the C-terminus of the MGC is devoid of any ligand binding ability and depended exclusively on the upstream binding modules to translate the ligand signals for generation of GMP.

Localization of the NCδ binding site within the core catalytic domain of ROS-GC1 created a new signaling model (Duda et al., 2012c). Now the Ca^2+^-bound NCδ direct interaction with the ROS-GC1 catalytic domain was sufficient for the ROS-GC activation.

### NC δ is also the Ca^2+^ sensor modulator of ANF-RGC

Because NCδ binds the catalytic module of ROS-GC and the sequence of this module is highly conserved in all members of the MGC family, it was natural to anticipate that the catalytic activity of all other members of the family is modulated by NCδ in a Ca^2+^-dependent manner. It was first tested with the ANF-RGC where the V^851^-L^872^ segment corresponding to the V^836^-L^857^ site of ROS-GC1 with the sequence identity of 68.2%. Accordingly, the experiments demonstrated that Ca^2+^-bound NCδ stimulated the catalytic activity of ANF-RGC in a dose-dependent fashion with an EC_50_ of 0.5 mM Ca^2+^ (Duda et al., 2012b). Neither ANF nor ATP were needed for the stimulation. Thus, the Ca^2+^-dependent stimulation was sufficient and was independent of the hormonal, ANF/ATP-dependent stimulation.

These results directed a new paradigm of the ANF-RGC signal transduction. Now, in addition to the ANF signal transduction, ANF-RGC was locked in with the NCδ-modulated Ca^2+^ signal transduction.

Myristoylation of NCδ was essential for activation of ANF-RGC. This finding rationalized the earlier results where Ca^2+^/non-myristoylated NCδ did not stimulate the ANF-GC activity (Kumar et al., 1999). Significantly, the myristoylation increased the ANF-RGC catalytic efficiency, *k*cat, from 6.5 ± 0.3 to 41.4 ± 0.5 pmol cyclic GMP/s. Myristoylated Ca^2+^-bound NCδ dimer forms a functional unit with the ANF-RGC dimer.

To determine the relationship between the Ca^2+^- and the ANF-modulated ANF-RGC signaling pathways, the ANF-RGC catalytic activity was monitored first in the presence of increasing concentrations of ANF (10^−11^–10^−6^ M) and constant 0.8 mM ATP, followed with the added 1 mM Ca^2+^ and 2 mM myristoylated NCδ. ANF/ATP caused 3.5-and 4.5-fold increase in the catalytic activity, and adding Ca^2+^-bound NCδ led to about 15-fold increase. Thus, the Ca^2+^-modulated and the ANF hormone-modulated pathways function independently.

To determine, and understand, the linkage of the biochemistry of NCδ modulation of ANF-RGC activity with cardiovascular physiology, a hemizygous NCδ knockout mouse, NCδ^+/^, was generated (Duda et al., 2012b). Immunohistochemical analyses demonstrated the co-presence of ANF-RGC and NCδ in adrenocortical zona glomerulosa (Takayanagi et al., 1987b; Sharma et al., 1989; Duda et al., 1991; Nakano et al., 1993; Rondeau et al., 1995). The particulate fraction of the adrenal gland from NCδ^+/−^ mice showed only 50% of the ANF-RGC activity of the NCδ^+/+^ mice; addition of exogenous NCδ and Ca^2+^ restored the total ANF-RGC activity.

#### Conclusion

The adrenal glands of mice embody the Ca^2+^/NCδ-modulated ANF-RGC signaling pathway. And, as anticipated, its functional activity is halved in the NCδ^+/−^ mice.

Physiologically, in adrenal glands the ANF/ANF-RGC hypotensive activity counterbalances the renin-angiotensin-aldosterone hypertensive activity. This results in lowering blood pressure (Aoki et al., 2000; Shi et al., 2001). As anticipated, in plasma of the NCδ^+/−^ mice the levels of aldosterone were about 27% higher than in the plasma of NCδ^+/+^ mice. But, significantly, the levels of corticosterone were similar in the plasma of both mice strains (Duda et al., 2014). Because aldosterone is synthesized in the adrenal glomerulosa cells, while corticosterone in the fasciculata cells, these results together with immunohistochemical analyses established co-expression of NCδ and ANF-RGC in the mouse adrenal glomerulosa cells, the cells that are physiologically linked with the blood pressure regulation (Duda et al., 2012b). Indeed, the systolic blood pressure of the NCδ^+/−^ mice was 38% higher than of the wild type mice, 127 vs. 92 mm Hg (Duda et al., 2012b).

Exposure of this novel Ca^2+^-modulated ANF-RGC signaling pathway presented an alternate mechanism to the control of the endocrine systems that prevent hypertension.

**Figure 8:**
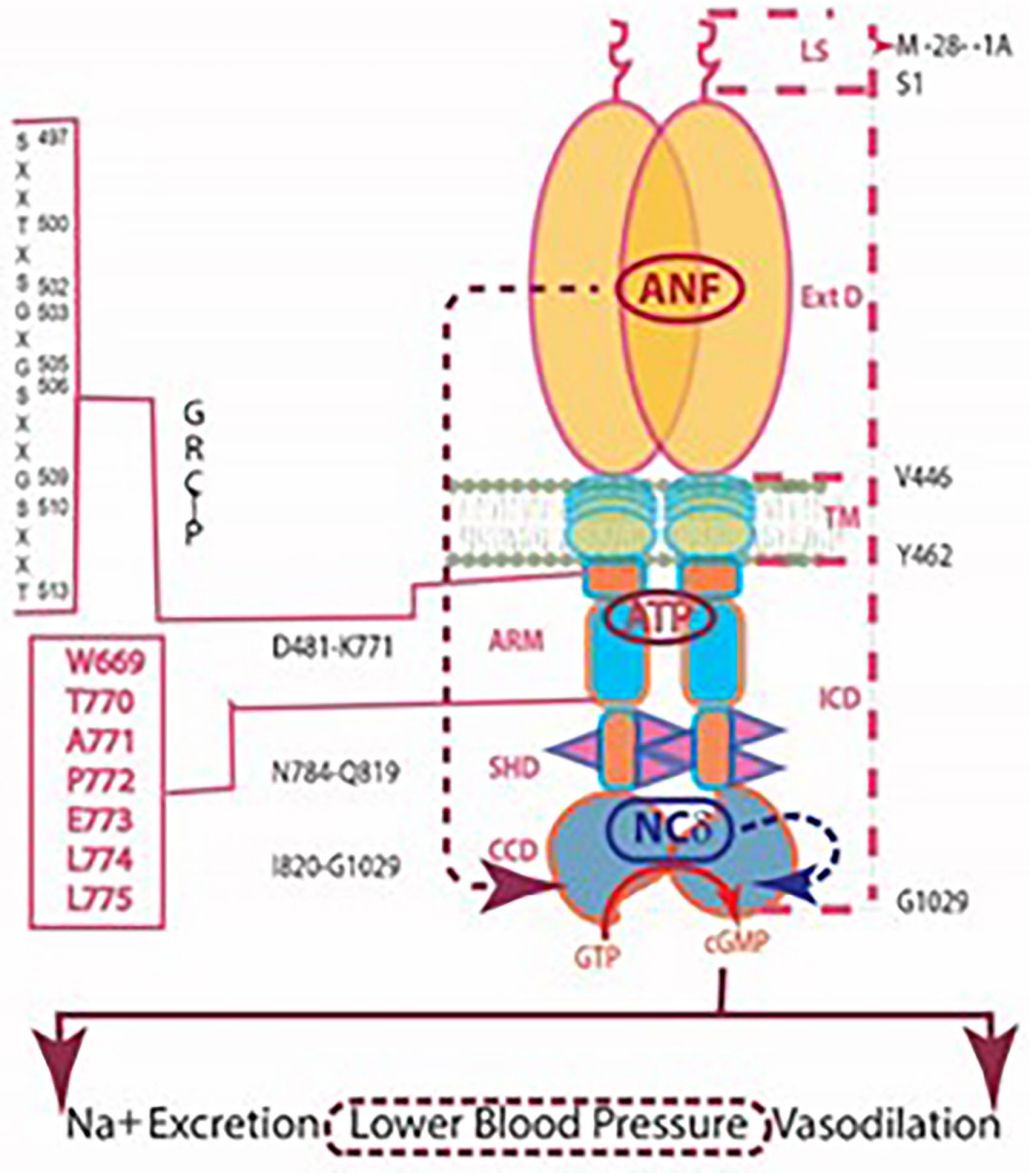
Independent signaling pathways of ANF/ATP and of NCδ. The trajectory of the ANF pathway (maroon dashed arrow) originates at the ExtD and passes through the TM, ARM and signaling helix domain (SHD) in its course to CCD. In contrast, the trajectory of the NCδ pathway (blue dashed arrow) lies within the CCD. The ANF-RGC dimer is thought to bind a dimer of NCδ, but only a single subunit is shown. Both pathways are the physiological regulators of the mouse blood pressure.

It also pointed that the new ANF-RGC transduction pathway is bimodal. It exists in some selected cells. There, when appropriate it controls the extracellular surface receptor signals and the intracellular Ca^2+^-modulated signals. This mechanism is illustrated in Figure 8.

Notably, following the same pattern, NCS and VILIP-1, two ANF-RGC Ca^2+^-binding proteins, were shown to bind ANF-RGC (Braunewell et al., 2001). As yet, there is no information, however, if they modulate ANF-RGC activity.

## Interlaced with Ca^2+^-sensing modulators, CO_2_/bicarbonate modulates a novel ROS-GC signaling pathway

In trail blazing studies, the MGC (ONE-GC) in olfactory neuro-epithelium was shown to sense atmospheric carbon dioxide (CO_2_; Hu et al., 2007; Guo et al., 2009). CO_2_ is the source of bicarbonate that signals ONE-RGC activation. The follow up observations of these authors were that the action of bicarbonate was ONE-GC-specific, since it did not affect the catalytic activity of the other recombinant forms of MGCs – ANF-RGC, CNP-RGC, STa-RGC and ROS-GCs (Guo et al., 2009; Sun et al., 2009).

Our group revisited this issue and found that bicarbonate targets directly the ONE-GC’s catalytic domain to stimulate it (Duda and Sharma, 2010). Since this structural domain is conserved, (85%) in all MGCs, we pursued the issue with recombinant ROS-GC1 with the following results.

**Figure 9:**
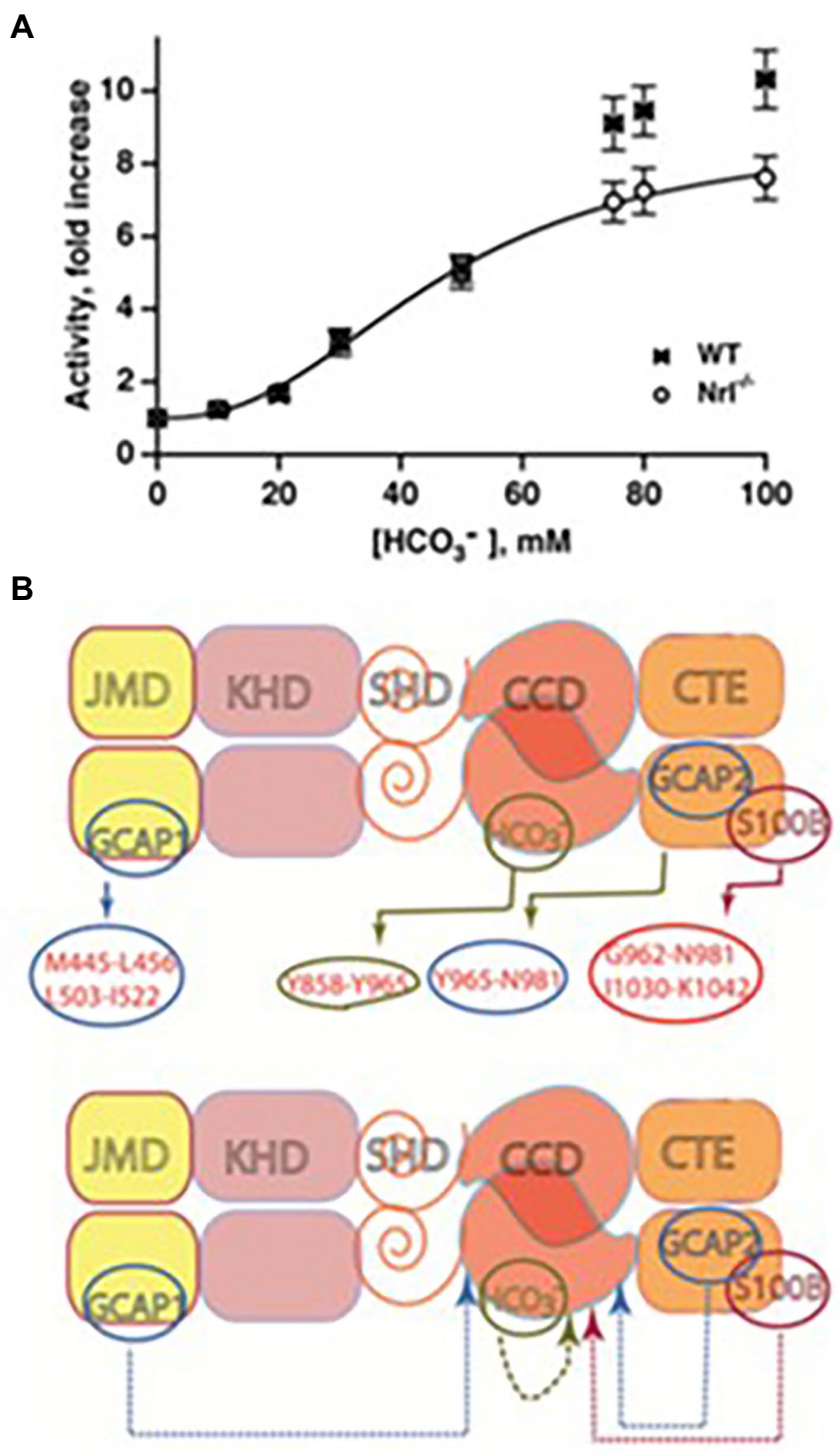
Bicarbonate modulation of ROS-GC activity. **(A)** Stimulation of ROS-GC in photoreceptor outer segment preparations from WT and neural retina leucine zipper transcription factor knock out (NRL−/−) mice. NRL−/− photoreceptors express ROS-GC1 and GCAP1 exclusively. The dependence of guanylate cyclase activity on bicarbonate is cooperative with an EC_50_ of 47 mM. The elevated activity at high bicarbonate concentration in WT outer segments is attributed to their additional expression of GCAP2. Error bars show SEM (Duda et al., 2015). **(B)** Ca^2+^-dependent and-independent modulators of ROS-GC1 activity. Upper panel: three Ca^2+^ sensor proteins – GCAP1, GCAP2, and S100B – and one Ca^2+^-independent modulator, bicarbonate, target individually the indicated domains within the intracellular portion of ROS-GC1. Lower panel: the targeted domains are specific switches all of which signal activation of the catalytic domain. The signaling pathways are indicated as dashed arrows.

The bicarbonate robustly stimulates the catalytic activity of recombinant bovine ROS-GC1 with an ED_50_ of 27 mM and a Hill coefficient of 2.8 and of ROS-GC2 with an ED_50_ of 39 mM and a Hill coefficient of 2.3 (Duda et al., 2015). The Hill coefficients >2 indicate that one or more bicarbonate molecules bind to each monomer. Similarly, bicarbonate stimulated the activities of ROS-GCs in the membranes of photoreceptor outer segments (Figure 9A).

To rule out the possibility that the observed increase in ROS-GC’s catalytic activity was through a pH dependent mechanism, the activity was tested over a range of pH from 7 to 9, and it remained constant (Duda et al., 2015).

The bicarbonate signaling of ROS-GC1 is independent of [Ca^2+^]_i_. Yet, bicarbonate synergizes with the Ca^2+^-sensors: GCAP1, GCAP2 and S100B to enhance Ca^2+^ modulation; the synergic effect is especially evident for GCAP2 (Duda et al., 2015, 2016; Figure 9B). The purpose of ROS-GCs activation in photoreceptors is to elevate the circulating current, to decrease sensitivity to flashes and to accelerate flash response recovery. Bicarbonate is a charged molecule and as such cannot freely pass through cell membranes. To gain access to ROS-GC in ROS it enters through the inner segment/synapse of intact rods. In contrast, it accesses ROS-GC1 of red-sensitive cones from the inner and outer segments (Makino et al., 2019).

Thereby, the findings clarified a large body of apparently controversial results on bicarbonate and cyclic GMP synthesis in retinal photoreceptors. They also provided a clue that bicarbonate signaling would be characteristic of most, if not all MGCs.

## Clinical implications

In human patients, a F^514^S mutation in ROS-GC1 was identified as the cause of Leber’s congenital amaurosis type1 (Perrault et al., 1996, 1999; Rozet et al., 2001). The mutation resulted in a 10-fold decrease in ROS-GC1 catalytic activity (Duda et al., 1999a,b); it also made the cyclase almost totally unresponsive to GCAP1 although the binding of GCAP1 to ROS-GC was not affected (Duda et al., 2016). It implies that the loss in GCAP1 modulation occurs at the signal transduction level and possibly involves one or more of the core catalytic residues: D^834^, E^874^, D^878^, R^925^, C^946^, and N^953^. In contrast, Ca^2+^-dependent modulations by GCAP2 and by S100B are unaffected by the F^514^S mutation (Duda et al., 1999a,b, 2016), even though the absolute achieved activities are reduced in all cases. The interaction of this disease-causing ROS-GC1 mutant with bicarbonate led to some insights into the intramolecular signaling pathways in ROS-GC1 catalytic activity.

Bicarbonate partially restores the basal as well as the GCAP2-and S100B-dependent activities of the F^514^S mutant but is ineffective for the deficit in GCAP1stimulation. The recuperative ability of bicarbonate indicates that it operates either downstream or independently of the F^514^S mutation. These findings support the earlier conclusion that the S100B-and GCAP2-targeted sites within ROS-GC1 overlap (Duda et al., 2002). Yet, both are distinct from the GCAP1-targeted site (Duda et al., 1996a, 2012c; Krishnan et al., 1998; Koch et al., 2010; Koch and Dell’Orco, 2013) although some indicate that GCAP2 binds to the same as GCAP1 site (Peshenko et al., 2015a,b).

At a clinical level, higher levels of bicarbonate could offer some relief for patients bearing the F^514^S-mutation by reinstating some basal and GCAP2-modulated guanylate cyclase activities in rods and cones. The mice stricken with the mutation would not be so lucky, however, as their cones express GCAP1 but not GCAP2 (Xu et al., 2013).

## ONE-GC senses odorants

Cloning of a MGC, GC-D, alternately termed ONE-GC from an olfactory cDNA library (Fulle et al., 1995) came at a time when G protein coupled receptors and the cyclic AMP signaling pathway were thought to be the sole components of the olfactory transduction (Buck, 1995; Belluscio et al., 1998; Breer, 2003; Lai et al., 2005). Even though an odorant for ONE-GC was not known, there were hints for a role of cyclic GMP in olfaction. *In situ* hybridization and immunocytochemistry results demonstrated that ONE-GC co-exists with PDE2, the phosphodiesterase, that hydrolyzes cyclic GMP as well as cyclic AMP and with a cyclic GMP gated ion channel (Fulle et al., 1995; Juilfs et al., 1997; Meyer et al., 2000). They are expressed only in a small subpopulation of neuroepithelial neurons that do not express any of the “standard” components: G*olf*, ACIII, PDE1C2, and the a3 and b1b subunits of the cyclic nucleotide gated ion channel. The ONE-GC/PDE2 expressing neurons project to specific “necklace glomeruli” of the olfactory bulb. Significantly, various anti-ONE-GC antibodies show more extensive labeling of olfactory neuroepithelial cilia suggesting the possible expression of more than one type of MGC and/or a subsidiary role for cGMP in a majority of the olfactory cells (Juilfs et al., 1997; Duda et al., 2001a).

To assess the possibility that a novel Ca^2+^-modulated MGC is present in the subpopulation of rat olfactory neuroepithelium cilia, our group cloned ONE-GC from the rat olfactory neuroepithelium cDNA library (Duda et al., 2001a). Sequence alignment revealed that the cloned cyclase is identical with the previously cloned GC-D (Juilfs et al., 1997), yet is only 47.9% and 47.6%, identical with, respectively, ROS-GC1 and ROS-GC2. A polyclonal antibody raised against its unique12 aminoacid C-terminal epitope recognizes neither ROS-GC1 nor ROS-GC2 indicating no immunological identity between these MGCs (Duda et al., 2001a). Thus, ONE-GC and GC-D are the same guanylate cyclase. Because ONE-GC resides in the olfactory neuroepithelium, the authors prefer the ONE-GC nomenclature over GC-D.

The ONE-GC system fulfils the requirements set forth to guarantee its role as a genuine, Ca^2+^-modulated odorant transducer (Duda et al., 2001a, 2004; Duda and Sharma, 2008; Sharma and Duda, 2010).

(1) It responds to uroguanylin in its natural environment. Uroguanylin is a very potent stimulus for ONE-GC expressing neurons (Leinders-Zufall et al., 2007), it binds the ONE-GC ExtD with an EC_50_ of 20 pM reaching plateau at 500 pM (Duda and Sharma, 2008). Although STa-RGC is a receptor for both uroguanylin and guanylin, ONE-GC is more selective and does not recognize guanylin; (2) Uroguanylin promotes the acquisition of food preferences in mice (Arakawa et al., 2013); (3) It responds to the odorant relatively quickly for an amplifying system; (4) ONE-GC is located within the ciliary membrane; (5) it is modulated by free Ca^2+^ with a *K*_1/2_ of 700 nM, generating responses similar to that of odorant; and (6) The Ca^2+^-responsive system can be reconstituted with recombinant myr-NCδ and ONE-GC.

ONE-GC binds NCδ through its M^880^-L^921^ segment that corresponds to the V^836^-L^857^ NCδ binding site in ROS-GC1, allowing direct access to the catalytic domain (Duda and Sharma, 2008). *In vivo* studies demonstrate that in resting cells, the NCδ-ONE-GC complex is solely located at the plasma membrane (Duda et al., 2004). The kinetic parameters of the Ca^2+^-bound NCδ binding to ONE-GC assessed by the surface plasmon resonance spectroscopy, are: *K*_D_ 2.8 × 10^−7^ M; *k*on = 5.7 × 10^3^ M^−1^ s^−1^; *k*off = 1.56 × 10^−3^ s^−1^.

**Figure 10:**
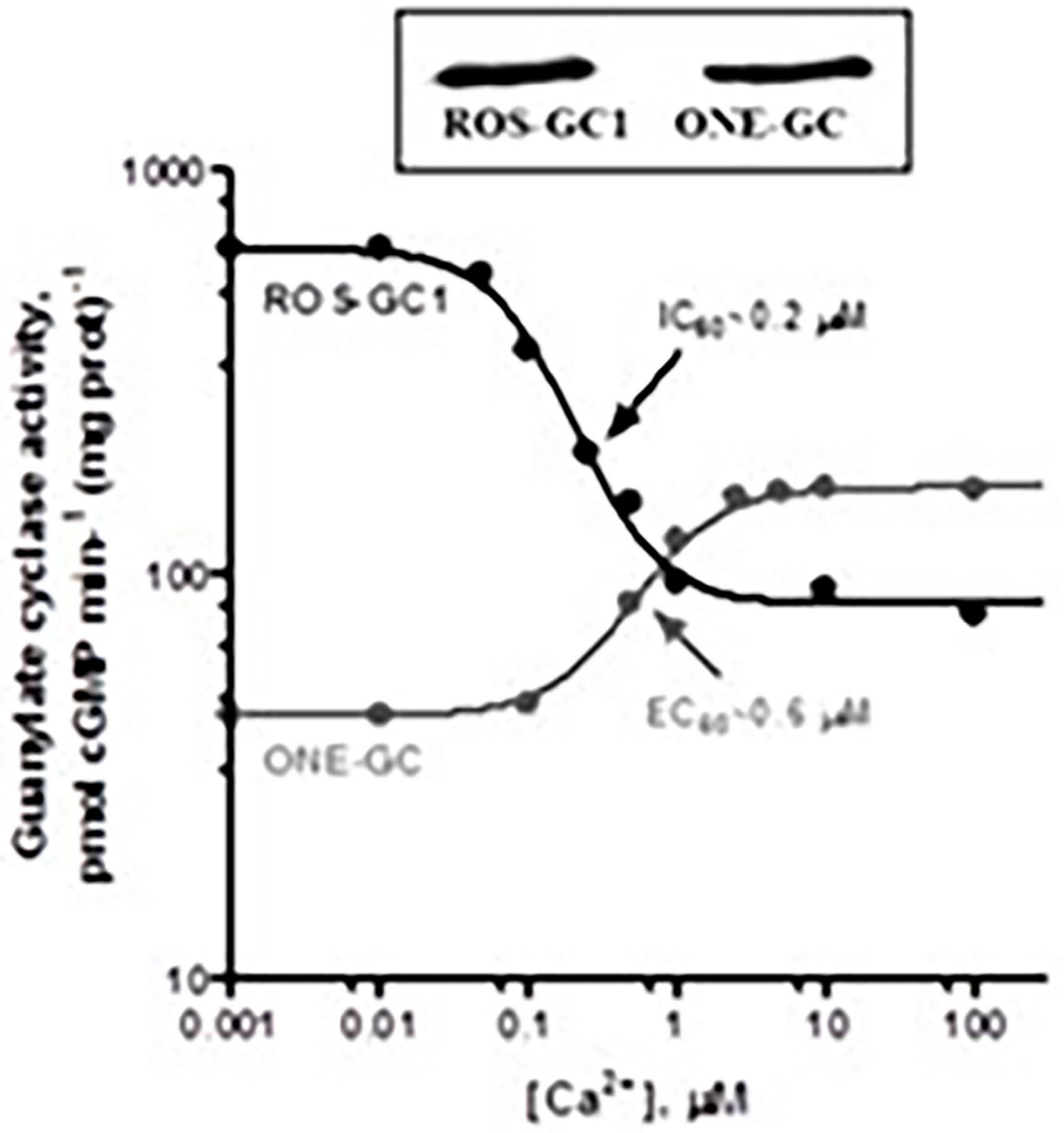
Antithetical Ca^2+^ modulation of ROS-GC1 and olfactory neuroepithelial guanylate cyclase (ONE-GC) activities by GCAP1. In the presence of GCAP1, the catalytic activity of recombinant ROS-GC1 decreases as Ca^2+^ is raised from 1 nM to100 mM, but the catalytic activity of recombinant ONE-GC increases. Western blots confirming ROS-GC1 and ONE-GC expression are shown above. Redrawn from Duda et al. (2012a).

Gene-knockout studies in mouse establish that in olfactory neuroepithelium another NCS, hippocalcin (Hpca) activates ONE-GC activity in the Ca^2+^ K_1/2_ range of 0.5–0.7 mM (Krishnan et al., 2009). Besides neurocalcin and Hpca, ONE-GC interacts with yet one more Ca^2+^ sensor, GCAP1 (Pertzev et al., 2010). The interaction was missed initially, because it is antithetical to that with ROS-GC in phototransduction. Instead of stimulating ONE-GC at the low nM range of free Ca^2+^ (i.e., in the Ca^2+^-free state), GCAP1 stimulates at the upper range of Ca^2+^ i.e., in the Ca^2+^ bound state (Duda et al., 2006, 2012a; Figure 10). To add to the trickery, stimulation of ONE-GC activity by GCAP1 occurs at an EC_50_ that is higher than the apparent IC_50_ for ROS-GC1 activity.

It was the first, and is to date, the only example of an NCS switching the directionality of its action with a change in binding partners. Biochemical assays with antibodies indicate that 35% of the total ONE-GC transduction activity is controlled by GCAP1, 27% by NCδ, and 38% by Hpca (Krishnan et al., 2009; reviewed in Sharma and Duda, 2010).

ONE-GC resembles the ANF-RGC in combining a surface hormone/odorant transduction with internal Ca^2+^ sensing. Yet, a primary sequence identity of only 28.1% with ANF-RGC but a sequence identity of 47.6% with ROS-GC makes it closer akin to the latter subfamily. Like photoreceptor ROS-GCs, ONE-GC is stimulated by bicarbonate (Guo et al., 2009; Sun et al., 2009). The target site of the bicarbonate signal resides within Y^922^-P^1028^ segment of the catalytic domain (Hu et al., 2007; Sun et al., 2009; Duda and Sharma, 2010).

## Hippocampus embodies ONE-GC linked with memory

The information that ONE-GC neurons sense the odorant and its perception occurs in the hippocampal neurons, paved the way for an exploration as to whether these neurons embody the ONE-GC-modulated signaling pathway? The issue was not fully decoded, yet pointed in that direction.

Because the Ca^2+^ sensor frequenin (Frq) is evolutionary the most ancient member of the NCSs family with homologs already expressed in yeast, it became the subject matter. The question was: Is Frq the Ca^2+^-sensor of ONE-GC?

Bovine Frq has a molecular mass of 22 kDa and shows a Ca^2+^-dependent electrophoretic mobility shift typical of the NCS family (Ladant, 1995). Its sequence is highly conserved within the evolutionary ladder with the identities of 100% with chicken (Nef et al., 1995; Olafsson et al., 1997; McFerran et al., 1999) rat, and human (Jeromin et al., 1999; Bourne et al., 2001); 98% with frog (Olafsson et al., 1995), 75% with *C. elegans* (De Castro et al., 1995), 72% with *Drosophila* (Pongs et al., 1993), and 60% with the yeast form (Hendricks et al., 1999). In common with the NCS family trait, it contained four EF-hands, yet only three – 2, 3 and 4 – are functional in binding Ca^2+^; and it is myristoylated.

Co-immunoprecipitation experiments determined that Frq and a ONE-GC-like MGC assemble in a complex in the neurons of bovine hippocampus (Fik-Rymarkiewicz et al., 2006). The complex lasts as long as Ca^2+^ is present and dissociates when Ca^2+^ is reduced or removed with EGTA. Remarkably, a very small fraction of Frq immunoprecipitates with the hippocampal MGC even in the presence of 5 mM EGTA. Hippocampal neurons respond to [Ca^2+^]_i_ with a five-fold increase in the MGC catalytic activity and a K_1/2_ for Ca^2+^ of 0.7 mM. The EC_50_ of hippocampal MGC for recombinant myr-Frq is 0.7 mM, and at saturating levels of Ca^2+^, the activity increases five-fold.

Recombinant myr-Frq stimulates the catalytic activity of recombinant ONE-GC 6-fold with an EC_50_ of 0.7 mM. It only marginally (1.5-fold) stimulates the rROS-GC1 activity, and fails to stimulate ROS-GC2.

The binding parameters between Frq and the M^836^-C^1110^ segment of ONE-GC were measured through surface plasmon resonance spectroscopy. They are: K_D_ = 0.43 mM; *k*on (the association rate constant) = 7.14 × 10^3^ M^−1^ s^−1^; *k*off (dissociation rate constant) = 3.04 × 10^−3^ s^−1^; and *K*A (equilibrium association constant) = 2.35 × 10^6^ M^−1^.

Thus, Frq is a soluble modulator rather than a subunit of the hippocampal MGC. Functionally, the existence of Frq in two forms, permanently bound to the hippocampal MGC (a minor fraction) and free in the cytoplasm of resting neurons (a larger fraction) serves to efficiently increase the Ca^2+^ requirement, extend the Ca^2+^ range and prolong the duration of the Ca^2+^ rise essential for stimulation of the hippocampal MGC activity.

Modulation of an MGC by myr-Frq suggests that evolutionary, Ca^2+^-sensors that stimulate MGCs at high Ca^2+^ probably preceded the GCAPs that stimulate at low Ca^2+^.

Additional studies demonstrate that hippocalcin, is also present in the hippocampal neurons, couples with ONE-GC, and modulates its activity in the Ca^2+^-dependent manner (Krishnan et al., 2009). Thus, it is potentially a second soluble Ca^2+^ modulator of the MGC.

Diverging from the interwoven concept of Ca^2+^-modulated ONE-GC, it is important to point out that the CNP-RGC expressed in hippocampal neurons may be locked in with the memory (Langub et al., 1995; Herman et al., 1996). Rats expressing a dominant-negative mutant of CNP-RGC lacking the intracellular cyclase catalytic domain show disturbances in long term potentiation and long term depression (Barmashenko et al., 2014). The studies on hippocampus has begun to unravel a role for MGCs for synaptic information storage and learning.

## Clued new frontiers of Ca^2+^-interlocked MGCS

At present there is growing evidence that MGCs have taken on in cellular signaling pathways throughout the body. A few examples are listed below.

### Pineal gland

The adrenergic receptor activity is linked to cyclic GMP synthesized by ROS-GC1 in a [Ca^2+^]_i_–dependent fashion (Venkataraman et al., 1998). Expression of both GCAP1 and the S100B in the pineal gland raises the possibility for the presence of two “Turn ON” Ca^2+^-modulated switches in the two separate subtypes of the pinealocytes (Venkataraman et al., 2000). One is GCAP1-and the other is S100B-modulated.

Pinealocytes also express ANF, CNP and CNP-RGC (Olcese et al., 1994; Middendorff et al., 1996). How they embody their signal transduction pathways is not yet explored?

### Gustatory epithelium

Expression of a Ca^2+^ modulated MGC transduction system in the anterior portion of the bovine gustatory epithelium, points out its role at the biochemical, molecular and functional levels in taste (Duda and Sharma, 2004). There are two components of the system: S100B, the Ca^2+^-sensor, and ROS-GC1, the transducer. ROS-GC1 and S100B co-immunoprecipitate indicating their physical interaction.

### Olfactory bulb

Biochemical and functional analyses of the rat olfactory bulb demonstrate the presence of GCAP1-modulated ROS-GC1 transduction system in its mitral cells. The system functions identically as in phototransduction model. GCAP1 senses lowering of free levels of Ca^2+^ and stimulates ROS-GC1 activity. The IC_50_ for Ca^2+^ is 70 nM. The bulb neurons do not express GCAP2, nor do they express ROS-GC2. The olfactory bulb receives neural input from the olfactory neurons and functions as a “processing unit” directing the information about odorants to the olfactory tract and to cortical centers of the brain for odorant perception (Duda et al., 2001b).

### Testes

There is ample evidence for the second messenger role of the cyclic GMP and for the presence of ANF-dependent ANF-RGC transduction system in testes. There, it appears they are involved in the varying physiological processes: sperm motility, development of testicular germ cells, relaxation of peritubular lamina propia cells, testosterone synthesis in Leydig cells and dilation of testicular blood vessels (Marala and Sharma, 1988; Singh et al., 1988; Garbers, 1989; Pandey and Singh, 1990; Armstrong et al., 1994; Middendorff et al., 1997, 2000; Pandey et al., 1999; Mourdjeva et al., 2001).

Biochemical studies demonstrate the presence of also ROS-GC1 transduction systems modulated by GCAP1 and S100B in the plasma membrane of bovine testes (Jankowska et al., 2007, 2014). These transduction components co-localize in bovine and human sperms (Jankowska and Warchol, 2010). A combination of molecular genetics, biochemistry and immunohistochemistry tools reveal the presence of NCδ in male gonads (Jankowska et al., 2014). Accordingly, GCAP1, S100B, and NCδ, sense and transmit Ca^2+^ signals to ROS-GC1 in this tissue. The transduction unit is present in all germinal cells of the human testes: spermatogonias, spermatocytes, and spermatids. NCδ appears to be the major (75%) and S100B the minor (25%) contributor of Ca^2+^ signaling as determined through mouse-KO models. Although the contribution of GCAP1 has not been determined yet, there is an evidence for its function in bovine and human spermatozoa (Jankowska and Warchol, 2010). The bimodal operation of MGC activity is evidenced by the results that its activity is minimal at 800 nM, increases with a decline in Ca^2+^ with an IC_50_ of 200 nM and increases again as Ca^2+^ is raised with an EC_50_ of 2 mM.

## A novel MGC (GC-G) is a thermosensor

Present in the certain temperature-sensitive neurons of the Grueneberg Ganglion in the mouse nasal cavity, GC-G is the latest discovered member of the MGC family (Schulz et al., 1998; Fleischer et al., 2009). At the time of this writing, its functional molecular mechanism is not defined. The limited information is provided below.

It has been cloned from rat and shown to be expressed in the selected Grueneberg Ganglion neurons. It is a thermosensor (Chao et al., 2015), reminiscent of MGCs encoded by gcy-8, gcy-18 and gcy-23 genes in *C. elegans* (Takeishi et al., 2016). It appears to have multiple functions such as sensing the predator odorant 2,4,5-trimethylthiazoline (Chao et al., 2015) and bicarbonate (Chao et al., 2010). Mild cooling of Grueneberg Ganglion neurons prompts ultrasound vocalization in pups, a behavior that is weakened in GC-G KO mice (Chao et al., 2015).

## Conclusion

The 1967, more than 52 years ago, kindled by my earlier pursuit that a common evolutionary link exists between the generation of plant steroids and the mammalian steroid hormones, I (RKS) entered the field of hormonal signal transduction and continued with it till now. In the ensuing years, a powerful group, including Teresa Duda was developed. It was dedicated to explore, generate and establish the new avenues of the hormonal signal transduction pathways. In this pursuit a pivotal finding was made that changed the cellular signal transduction field. It was the discovery of the MGC signal transduction pathway/s.

**Figure 11:**
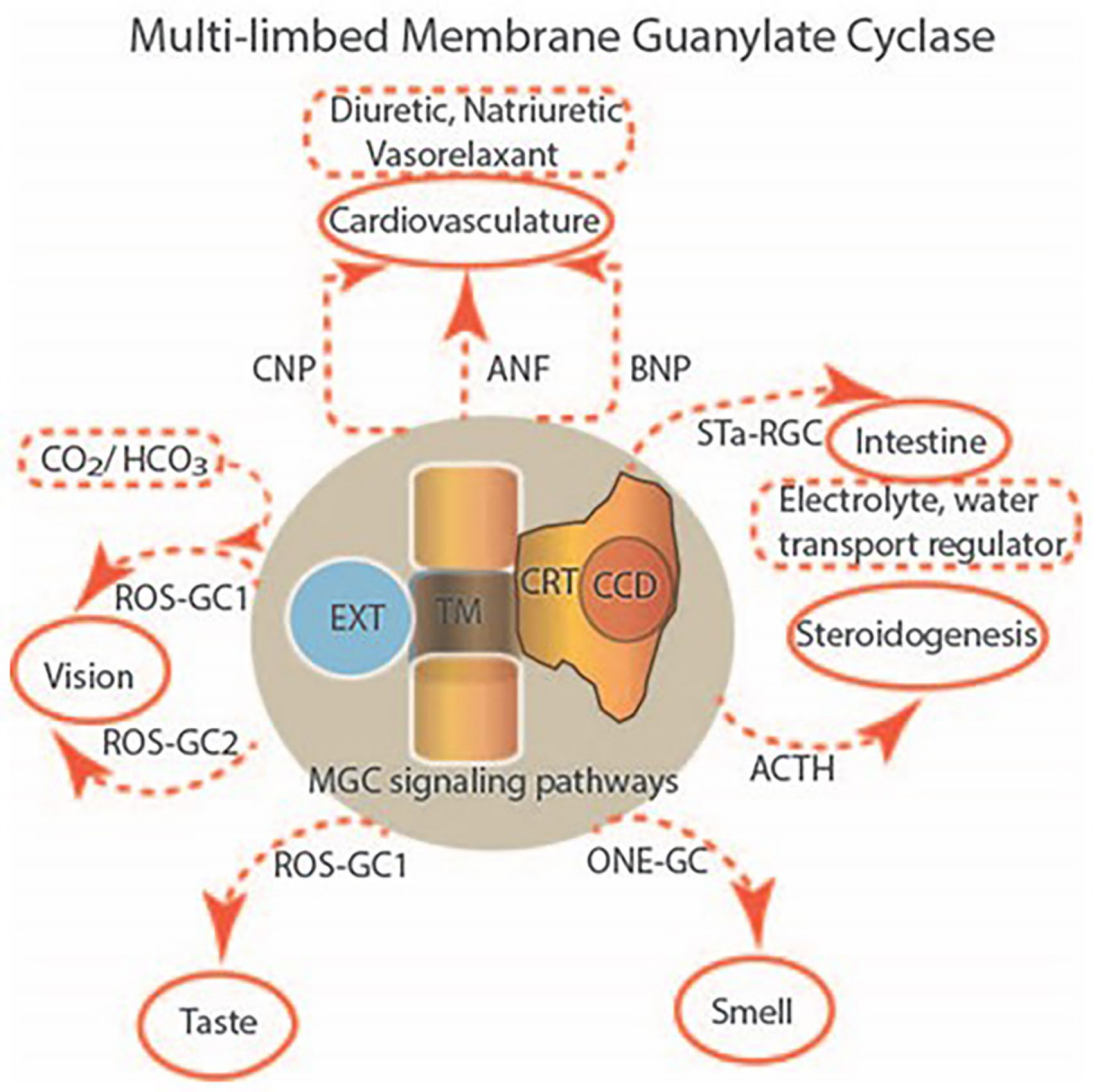
Multilimbed membrane guanylate cyclase. In mammals, membrane guanylate cyclase signaling is encoded by seven distinct genes. Through their expression in seven subfamily forms, they perform multi-limbed functions. The surface receptor subfamily, detects hormones and other extracellular chemicals, translate the signal across their plasma membrane domains and by way of their second messenger, cyclic GMP, regulate an intracellular pathway to impact physiology. Sequentially, other similar signaling networks, depicted in the figure, were discovered. Collectively, besides steroidogenesis, they control the physiology of the cardiovasculature, sensory neurons: vision, taste and smell, the intestine and skeletal growth. Uniquely designed, all forms are single-pass transmembrane proteins, embedded with an EXT, TM, CRM and CCD topography. Abbreviations: EXT, extracellular; TM, transmembrane; CRM, catalytic regulatory module; CCD, catalytic core domain.

**Figure 12:**
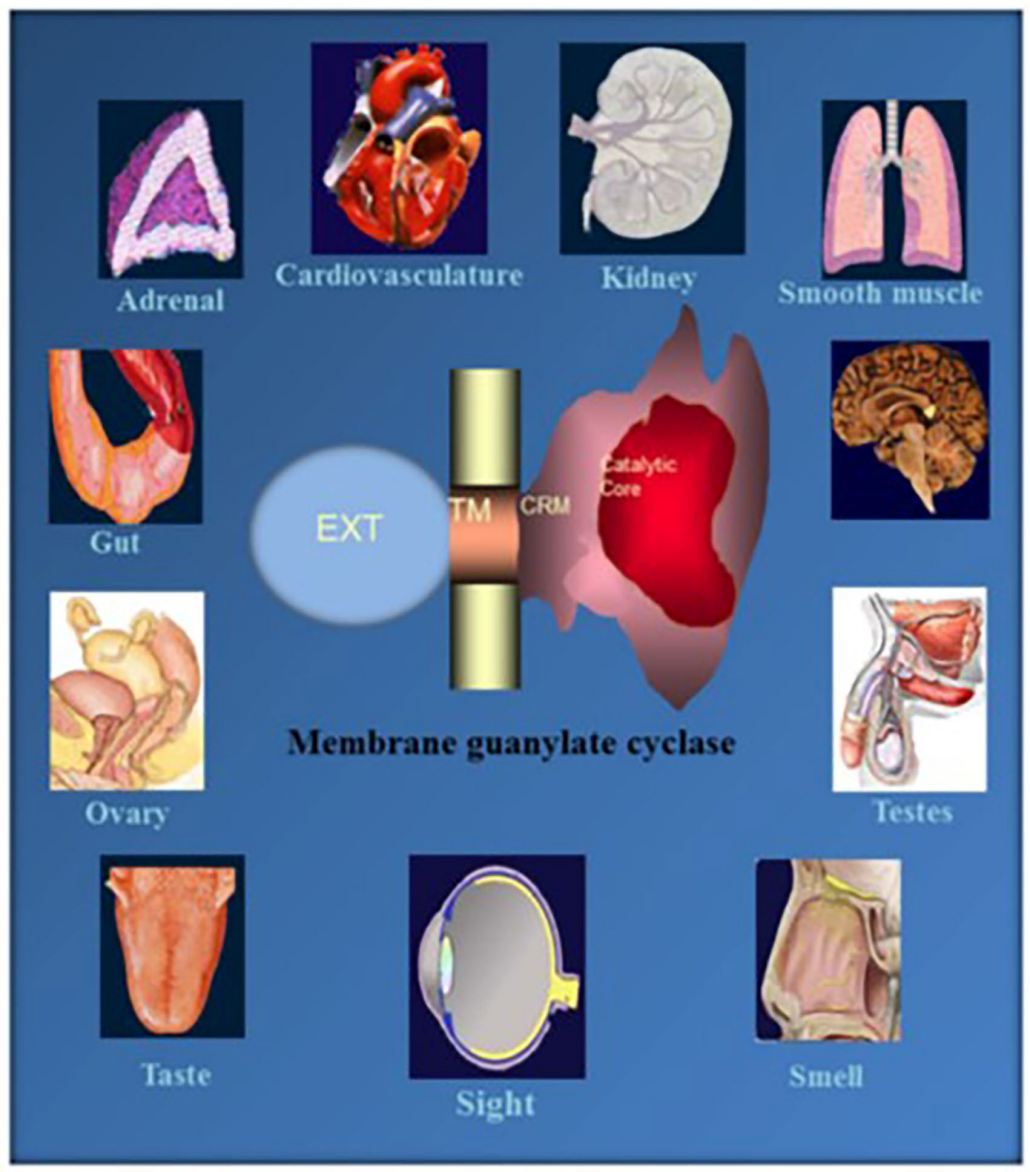
Ubiquitous expression of MGC signaling systems. MGCs is a multi-switching cyclic GMP generating machine linked with the physiology of cardio-vasculature, smooth muscle relaxation, sensory transduction, neuronal plasticity and memory in mammalian neurosensory, endocrine and peripheral tissues. (Reproduced from Sharma et al., 2015).

The first foundational brick demonstrated that this system is embodied in the rat adrenocortical carcinoma 494 cells, and it is epinephrine-specific (Perchellet and Sharma, 1980; Shanker and Sharma, 1980). This monograph recounts its evolutionary tale. Its painstaking analysis, its growth from obscurity to its present position and its emergence as a dominant cellular signal transducer. Generating, its intracellular second messenger, cyclic GMP, it judiciously controls the, biochemistry and molecular biology of the innumerable physiological processes (Figures 11, 12).

What Are Its coded molecular features that make It achieve such complex tasks? (1) A modular structure. It makes the MGC enormously elastic and multimodal. Unlike its predecessor three component second messenger systems, adenylate cyclase and IP_3_, the MGC is composed of a single unit. It is a trans-membrane-spanning protein. It functions both as surface receptor and the signal transducer. (2) All MGCs contain the ExtD. Except for ROS-GC1 and ROS-GC2, it is the binding domain of the hormones. Its role in ROS-GC1 and ROS-GC2 is unknown. (3) A few MGCs embody subunits. The subunits join to an intracellular domain (ICD) and bestow the MGC Ca^2+^ sensitivity. (4) The kinship between Ca^2+^ and MGC’s catalytic activity is irregular. (5) ANF-RGC. High Ca^2+^ stimulates its catalytic activity without ligand binding. Yet, ROS-GC1 and ONE-GC can function as bimodal Ca^2+^ switches. Its catalytic activity fluctuates. It is maximal as Ca^2+^ levels lower, it wanes at midrange [Ca^2+^]_I_ and rises once again as [Ca^2+^] reaches its peak level. The reason for these different modes trace to the nature of the Ca^2+^ sensor involved – GCAPs, S100B, neurocalcin δ, hippocalcin, and frequenin. (6) In an amazing arrangement, the function of GCAP1 is its MGC partner-dependent; at low Ca^2+^, it stimulates ROS-GCs and inhibits them at high Ca^2+^. Yet, it stimulates ONE-GC at high Ca^2+^. (7) ROS-GC1 has the ability to link with varied forms of Ca^2+^ sensors (Figure 11 of Sharma et al., 2016), in part because of its CTE. (8) In contrast, ROS-GC2 is discriminatory, despite it possessing a CTE. Intriguingly, the CTE in STa-RGC points out additional functions this structure performs. (9) The conserved CCD dimer receives signals generated by both the C-terminal and N-terminal modules. (10) Extraordinarily, the CCD is also subject to direct tuning by Ca^2+^/neurocalcin δ and bicarbonate. (11) With conservation, the CCD structure of every MGC responds to the bicarbonate signals. The response of ROS-GC to bicarbonate and Ca^2+^-modulated GCAPs and S100B in combination exceeds the sum of the individual modulations. Thus, bicarbonate provides a means to amplify the transductions. (11) Probably, the bicarbonate signaling can amplify transduction of orthosteric ligand binding. (12) The modulation and regulation by phosphorylation plays a critical part in hormone receptor MGCs.

## Author contributions

Both authors have made a substantial, direct, intellectual and equal contribution to the work and approved it for publication.

## Conflict of interest

The authors declare that the research was conducted in the absence of any commercial or financial relationships that could be construed as a potential conflict of interest.

## Publisher’s note

All claims expressed in this article are solely those of the authors and do not necessarily represent those of their affiliated organizations, or those of the publisher, the editors and the reviewers. Any product that may be evaluated in this article, or claim that may be made by its manufacturer, is not guaranteed or endorsed by the publisher.
